# Diversity of Sex Chromosomes in Vertebrates: Six Novel Sex Chromosomes in Basal Haplochromines (Teleostei: Cichlidae)

**DOI:** 10.1093/gbe/evae152

**Published:** 2024-07-29

**Authors:** Kristen A Behrens, Stephan Koblmueller, Thomas D Kocher

**Affiliations:** Department of Biology, University of Maryland, College Park, MD 20742, USA; Institute of Biology, University of Graz, 8010 Graz, Austria; Department of Biology, University of Maryland, College Park, MD 20742, USA

**Keywords:** African cichlids, sex chromosome turnover, *Pseudocrenilabrus*

## Abstract

African cichlid fishes are known for their high rates of phenotypic evolution. A rapid rate of diversification is apparent also in the diversity of their sex chromosomes. To date, sex determiners have been identified on 18 of 22 chromosomes in the standard karyotype. Here, we use whole-genome sequencing to characterize the sex chromosomes of seven populations of basal haplochromines, focusing on the genus *Pseudocrenilabrus*. We identify six new sex chromosome systems, including the first report of a cichlid sex–determining system on linkage group 12. We then quantify the rates and patterns of sex chromosome turnover in this clade. Finally, we test whether some autosomes become sex chromosomes in East African cichlids more often than expected by chance.

**Significance**

It has been suggested that a relatively small number of chromosomes can become sex chromosomes in vertebrates, but this hypothesis has rarely been subjected to statistical analysis. Here, we add to the growing list of sex-determining loci in African cichlid fishes and then show that the repeated emergence of sex-determining loci on particular chromosomes is consistent with Poisson expectations. Our results demonstrate that most autosomes can become sex chromosomes in this lineage.

## Introduction

In an influential review, Marshall Graves and Peichel (2010) asked why the same autosomes were repeatedly used as sex chromosomes in different vertebrate lineages. Most readers have focused on the two hypotheses offered in their introduction. However, in their discussion, they offered three hypotheses for why the same chromosomes might be used repeatedly.

The first hypothesis was that the similarities might be due to shared retention of ancestral sex chromosomes from a common ancestor. Certainly, this is the case for the widespread use of SRY in mammals. A similar case was made for DMRT1 in birds and snakes, but subsequent studies have shown that snakes do not all share a common ZW system (Gamble et al. 2017). Furthermore, a growing number of studies have revealed a great diversity of sex chromosome systems in reptiles and fishes (Pinto et al. 2022; Zhu et al. 2022; Kitano et al. 2024) which could not have been inherited from a common ancestor.

The second hypothesis, the limited option hypothesis, was that some autosomes might be reused as a sex chromosome because they carry one of a limited number of genes that can take control of the sex-determining pathway. At the time, only a short list of genes, especially members of the *sox* and *dmrt* families, had been implicated as sex determiners. Although Marshall Graves and Peichel hinted that a diversity of genetic triggers might exist in fishes, frogs, and reptiles, the thrust of their review was that only a few genes interacting directly with the core machinery of sex differentiation could take on the role of sex determiner.

The third hypothesis was that some autosomes might carry an unusual amount of sexually antagonistic variation that might recruit a nearby sex-determining gene. Marshall Graves and Peichel highlight the frequent linkage of sex determiners with pigmentation loci in fishes, which might have arisen under selection for sexually antagonistic nuptial coloration (Lindholm and Breden 2002; Roberts et al. 2009). We can imagine a variety of additional hypotheses for a nonrandom pattern of recruitment. For example, some chromosome segments might have unusually low levels of recombination that might favor the association of new sex determiners with selectively advantageous variation (Rifkin et al. 2021).

Here, we focus on whether the pattern assumed by Marshall Graves and Peichel actually exists. Are some chromosomes used as sex chromosomes more often than others? There is reason for doubt. Recent reviews have tabulated a growing list of genes suggested to be sex determiners in a variety of vertebrates (Curzon et al. 2023; Kitano et al. 2024). This anecdotal accumulation of candidate genes has a number of inherent ascertainment biases. For example, when a previously known sex gene is found within a sex-linked interval, it is quickly assumed to be the causative locus, often without significant functional data. The standards of proof for demonstrating a causative effect of a particular candidate gene on sex determination have not been codified. On the other hand, when the usual suspects are not found in the interval, often no candidate gene is reported, even though this represents prima facie evidence for a new player in vertebrate sex determination. Often, the region of differentiation between the sex chromosomes is so large that it encompasses many potential candidate genes and thus is not included in the reviews. Furthermore, these reviews have been structured to tabulate the use of individual genes, not chromosome segments. We are aware of only one study that has performed a formal statistical analysis of the use of chromosome segments (Kratochvíl et al. 2021). Their sample included 27 independent sex chromosome origins in amniotes and found only weak support for the nonrandom recruitment of particular syntenic blocks. We reasoned that a larger sample might be helpful in determining whether some chromosomes are used more often than others.

Cichlid fishes (Cichlidae) have radiated into more than 1,500 species in East Africa over the last 25 million years. This clade has become a model system for studying a variety of evolutionary processes (Kocher 2004; Santos et al. 2023). Research over the past 20 years has begun to reveal the great diversity of sex chromosomes in African cichlids. The first sex determiners in oreochromine cichlids were mapped to linkage group (LG) 1 (Lee et al. 2003) and LG3 (Lee et al. 2004). A third locus was mapped to LG23 (Shirak et al. 2006) and eventually associated with a duplication of anti-Müllerian hormone (*amh*) (Eshel et al. 2014; Li et al. 2015).

Many more sex chromosomes have been identified in the radiation of cichlids that gave rise to the species flocks in the African Great Lakes. In Lake Malawi, sex was mapped to an XY locus on LG7 (Ser et al. 2010; Munby et al. 2021), a ZW locus associated with the orange-blotch color morph on LG5 (Roberts et al. 2009), and a W locus on an unpaired B chromosome (Clark and Kocher 2019). In Lake Victoria, sex loci have been found on B chromosomes (Yoshida et al. 2011), LG2 and LG5 (Kudo et al. 2015), LG23 (Feulner et al. 2018), LG9 (Feller et al. 2021), and LG16 (Kocher et al. 2022). In Lake Tanganyika, sex has been mapped to at least 12 different LGs (Gammerdinger et al. 2018; El Taher et al. 2021; Behrens et al. 2022; 2024). Additional sex chromosomes have been identified in riverine haplochromines, including evidence for three systems in *Astatotilapia burtoni* (Böhne et al. 2016; Roberts et al. 2016). To date, 18 of the 23 chromosomes have been identified as sex chromosomes in one species or another. The overall rate of sex chromosome turnover in this lineage is at least 0.186 turnovers per million years (El Taher et al. 2021; Behrens et al. 2024).

**Fig. 1.**
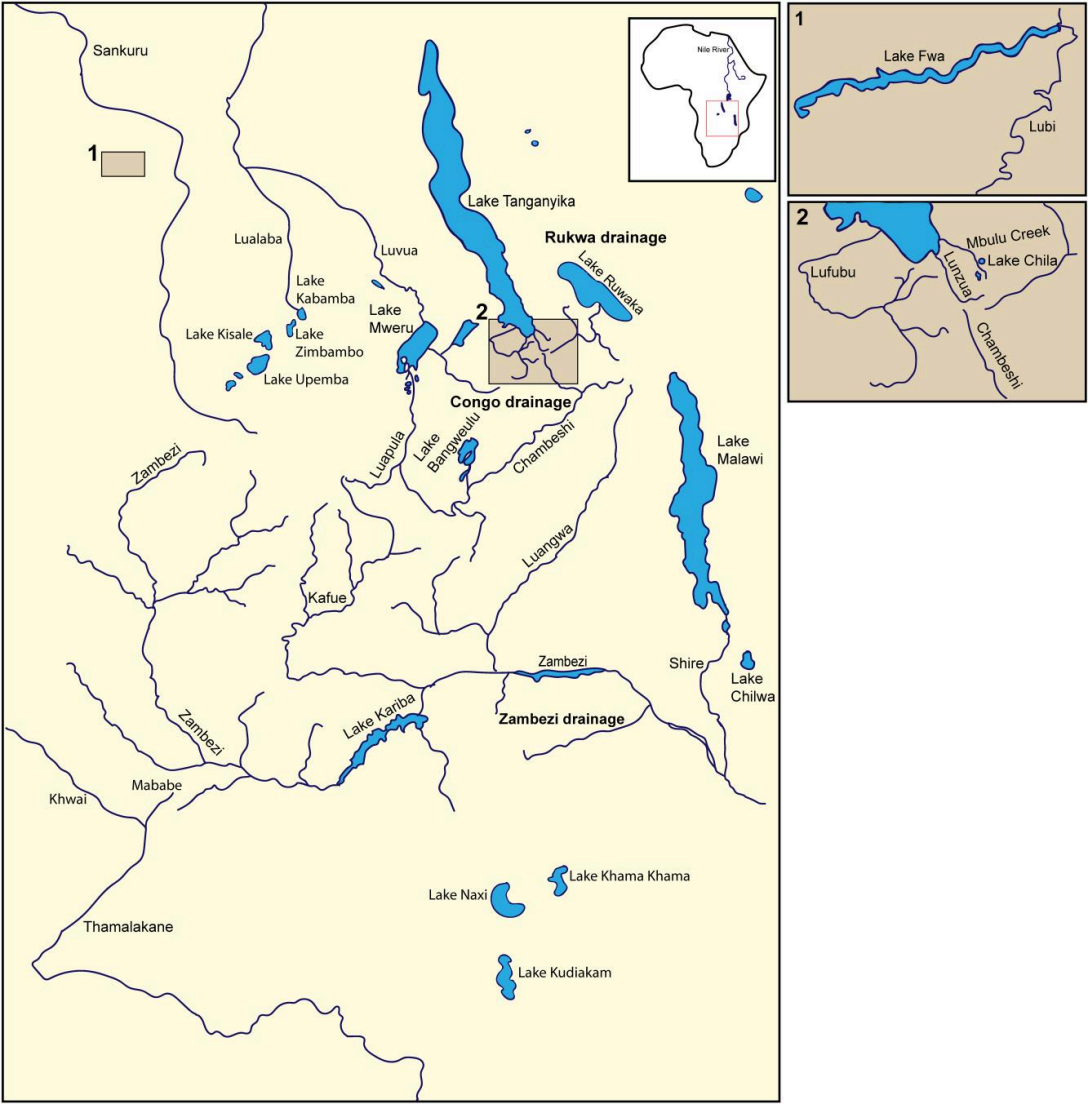
Geographic context of the basal haplochromines. Map depicting approximate locations of rivers and lakes relevant to this study, adapted from Egger et al. (2015).

The sister group of the modern haplochromines is composed of two main clades that are less species rich: (i) the *Serranochromis*-like cichlids that occur in Central, Eastern, and Southern Africa and (ii) the *Pseudocrenilabrus*-like cichlids that are found across large parts of Africa from Northern Egypt over Central and Eastern Africa to South Africa. In lacustrine environments outside the core distribution of the modern haplochromines, these lineages repeatedly formed small adaptive radiations (Roberts and Kullander 1994; Joyce et al. 2005; Meier et al. 2019). Of particular interest is the genus *Pseudocrenilabrus*, which was previously shown to have evolved different sex determination systems in different populations/species (Böhne et al. 2019). Currently, this genus comprises four valid species, whose most recent common ancestor dates back ∼2 MY (Koblmüller, Schliewen, et al. 2008; Irisarri et al. 2018; Meier et al. 2019; Schedel et al. 2019). These described species are allopatrically distributed from Northern Egypt to South Africa, but do not reflect the true diversity in this genus (Katongo et al. 2005; Koblmüller, Sefc, et al. 2008, 2012; Egger et al. 2015; Meier et al. 2019). *Pseudocrenilabrus* are predominantly found in small creeks and flood plains, where they are impacted by seasonal changes that affect the water level. Thus, populations are separated and rejoined at various points or come into secondary contact via river capture or flooding events (Koblmüller, Sefc, et al. 2008, 2012; Egger et al. 2015). Indeed, hybridization among previously allopatric *Pseudocrenilabrus* lineages facilitated an adaptive radiation in Lake Mweru (Meier et al. 2019). The geographic region inhabited by these species/populations is shown in Fig. 1.

Previous work studied sex chromosomes in two populations of *Pseudocrenilabrus philander* in Eastern Zambia, just south of Lake Tanganyika (Böhne et al. 2019). The population in Lake Chila was found to have an XY-LG7 system that was not related to the XY-LG7 segregating in Lake Malawi. This system was not detected in the Mbulu Creek population, nor in any of the *Pseudocrenilabrus* complex species examined for cosegregation of two Y-linked markers from the Lake Chila population (Böhne et al. 2019). The absence of the XY-LG7 system in Mbulu Creek is surprising, as Mbulu Creek is connected to Lake Chila via the lake's outflow. Additionally, another member of the *Pseudocrenilabrus* clade, *Orthochromis indermauri*, has an XY-LG19 system which has been suggested to share an origin with the XY-LG19 system in *Tropheus* (Gammerdinger et al. 2018; El Taher et al. 2021) and therefore may be ancestral to *Pseudocrenilabrus*. Together, these observations suggested that there might be undiscovered sex chromosomes systems in the *Pseudocrenilabrus* complex.

In the present study, we expanded the sampling of *Pseudocrenilabrus* to include two additional populations of *P. philander*, two additional species of *Pseudocrenilabrus*, and the two recognized subspecies of *Pseudocrenilabrus multicolor* (*multicolor multicolor* and *multicolor victoriae*). We also sampled *Lufubuchromis relictus*, a more distantly related species in the *Pseudocrenilabrus* clade, and a single species from the *Serranochromis*-like clade, *Thoracochromis callichromus*. We used whole-genome sequencing to analyze sequence differentiation between pools of male and female DNA and identify the sex chromosomes in each population. Our goals were to (i) identify the ancestral state of sex determination in the common ancestor of the haplochromine lineage, (ii) quantify the rates of sex chromosome turnover and differentiation, and (iii) statistically test whether autosomes have an equal chance of being recruited to the role of sex chromosome in the East African cichlid lineage.

## Results

In the seven new populations studied, we identified six unique sex chromosome systems involving six different chromosome pairs. We discuss the results for each population in separate sections below.

### *P. philander* (Thamalakane River)

**Fig. 2.**
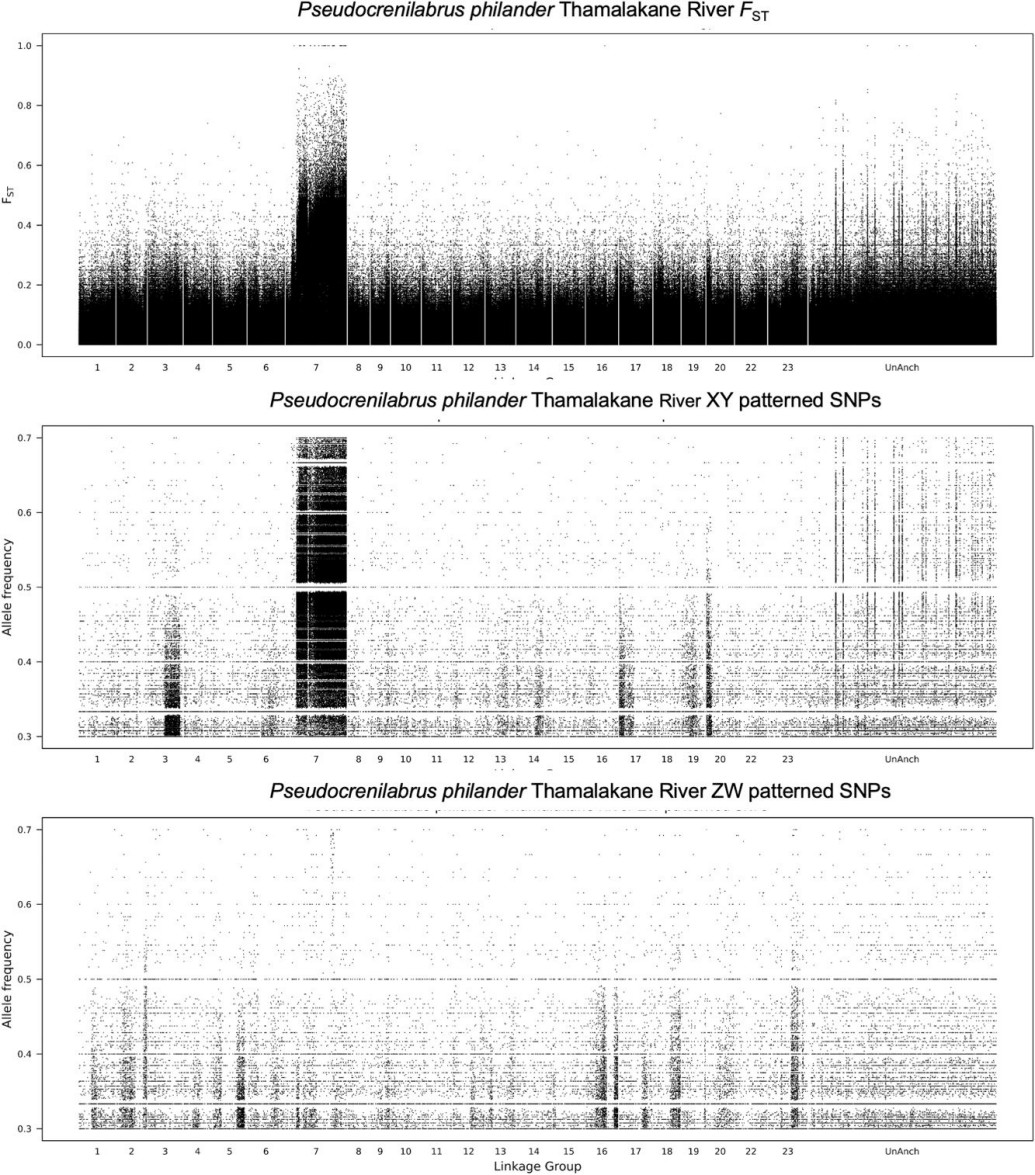
Differentiation between male and female *P. philander* “Thamalakane River.” The top panel plots *F*_ST_-quantifying allele frequency differences between the male and female DNA pools. The middle panel shows the frequency of male-patterned (Y) alleles, and the bottom panel shows the frequency of female-patterned (W) alleles.

The *F*_ST_ plot for this population shows strong signal encompassing most of LG7 (Fig. 2). The vast majority of sex-patterned single nucleotide polymorphisms (SNPs) (defined as SNPs with frequency < 0.1 in one sex and between 0.3 and 0.7 in the other sex) indicate a male heterogametic (XY) system. An analysis of sex-patterned SNPs in 100 kb windows across the genome revealed that all 78 of the top 1% of windows showed an XY pattern on LG7 (supplementary table S1, Supplementary Material online). The sex-patterned SNPs in the unanchored contigs of the *Metriaclima zebra* UMD2a assembly map onto LG7 when the more contiguous *Oreochromis niloticus* UMDNMBU assembly is used as a reference.

A more detailed examination of the differentiation on LG7 reveals at least two clear strata with different densities of sex-patterned SNPs (supplementary fig. S1, Supplementary Material online). It is not possible to suggest candidate genes for this system because the oldest evolutionary stratum encompasses approximately half of the chromosome. However, the oldest stratum does include gonadal somatic cell–derived factor (*gsdf*).

### *P. philander* (Lake Chila and Mbulu Creek)

We compared our results for the *P. philander* Thamalakane River population with the previously published data on populations of *P. philander* from Lake Chila and the adjacent Mbulu Creek in Zambia (Böhne et al. 2019). The *F*_ST_ plots for these populations are noisy, either because these populations have a small *N*_e_ or because only a small number of individuals were sequenced from each population (6M, 6F from Lake Chila and 4M, 3F from Mbulu Creek).

Nevertheless, in Lake Chila, there is a strong XY signal on LG7 (supplementary fig. S2, Supplementary Material online). The pattern of differentiation looks very similar to that previously reported on the *O. niloticus* reference (Böhne et al. 2019). All 78 of the top 1% sex-patterned windows are XY on LG7 (supplementary table S2, Supplementary Material online).

The plots for the Mbulu Creek sample do not show an obvious sex signal (supplementary fig. S3, Supplementary Material online). The Kruskal–Wallis (KW) test shows heterogeneity of sex-patterned SNPs among chromosomes (supplementary table S3, Supplementary Material online). The strongest signals are XY signals on LG2, LG3, LG4, LG5, and LG6. Although the Dunn's test suggests the greatest differentiation on LG4, these signals likely arise from identity by descent in this small sample from a population which may also have a small *N*_e_. However, the absence of signal on LG7 is surprising, as the strongly differentiated XY system found in Lake Chila should have been easily detected in the sample from its outflow, Mbulu Creek.

### *P. philander* (Lake Mweru)

**Fig. 3.**
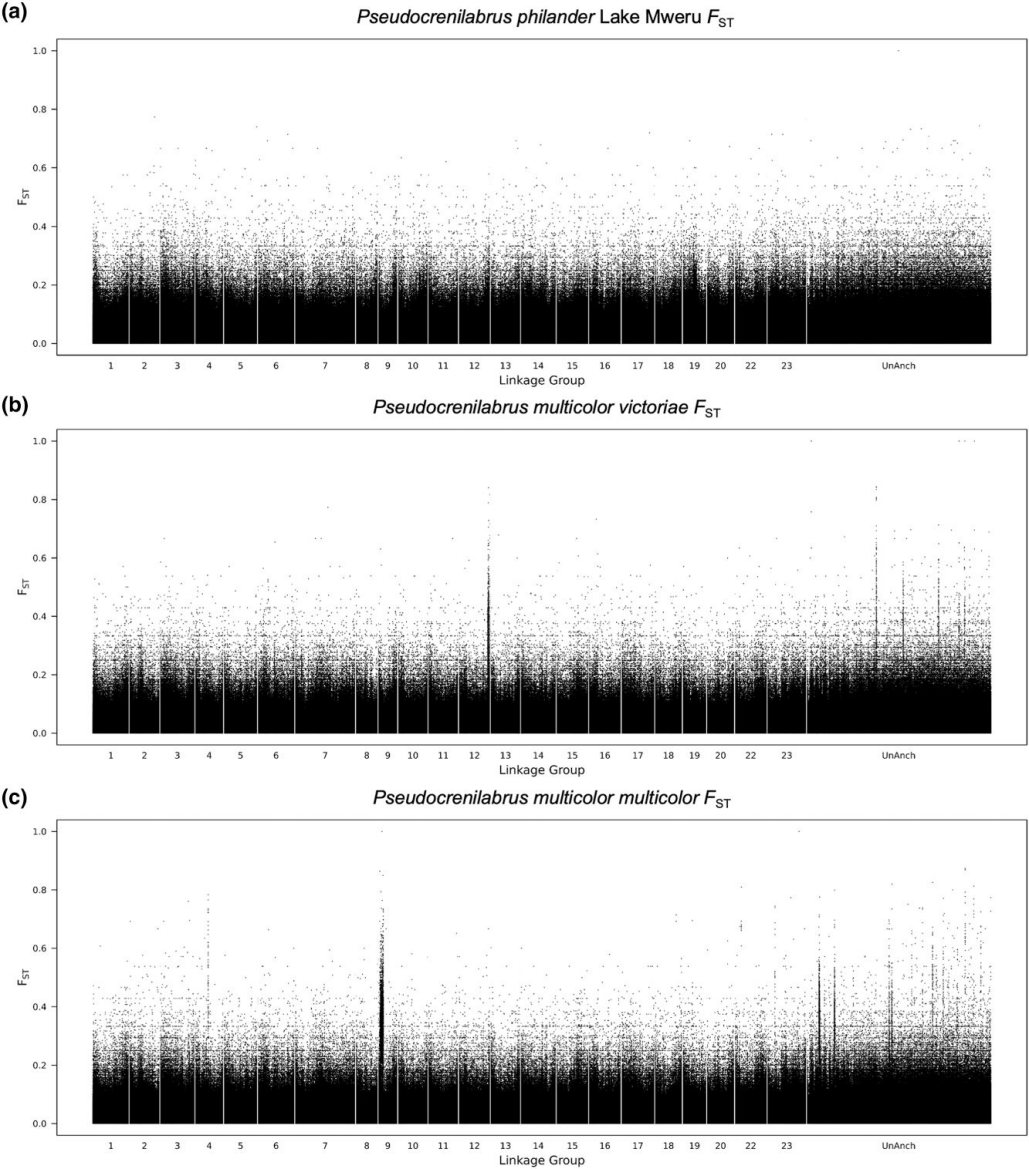
Whole-genome *F*_ST_ plots on the *M. zebra* UMD2a reference for a) *P. philander* “Lake Mweru,” b) *P. multicolor victoriae*, and c) *P. multicolor multicolor*.

There is no obvious signal in the *F*_ST_ plot for the Lake Mweru population (Fig. 3a). However, the top 1% windows provide strong support (36 of the 96 windows) for a ZW system on LG23 (supplementary table S4 and fig. S4, Supplementary Material online). The differentiated region includes *amh*, which has been implicated in sex determination in several other fishes. However, when we examined the read mappings in Integrative Genomics Viewer (IGV), we could find no evidence of sequence coverage differences, copy number variation, or other structural rearrangements that would indicate that *amh* has been recruited as the sex-determining gene.

### P. multicolor victoriae

The *F*_ST_ plot shows a narrow peak of XY-patterned differentiation on LG12 (Fig. 3b). Peaks in the unanchored contigs coalesce onto LG12 in the more contiguous assembly of *O. niloticus* (supplementary fig. S5, Supplementary Material online). The analysis of the top 1% windows strongly supports an XY system on LG12 (19 of the 89 top 1% windows; supplementary table S5, Supplementary Material online). This is the first identification of a sex-determining locus on LG12 in cichlids.

The differentiated region appears to be a single stratum which extends over approximately 2 Mb. Within this region, there are a number of candidate genes for sex determination, including *dmrt1*, *dmrt2*, *dmrt3*, and *mapk1*. No structural changes were noted that would support one of these candidate genes as the sex determiner.

### P. multicolor multicolor

The *F*_ST_ plot shows a narrow peak on LG9 (Fig. 3c). Signals on unanchored contigs of the *M. zebra* (UMD2a) assembly are coalesced into a single LG9 peak when the reads are mapped on the more contiguous *O. niloticus* (UMDNMBU) assembly (supplementary fig. S6, Supplementary Material online). The analysis of sex-patterned SNPs indicates a ZW system. The analysis of the top 1% windows shows 43 ZW windows on LG9 (supplementary table S6, Supplementary Material online).

A detailed examination of the differentiation of LG9 reveals a single stratum about 3.5 Mb in length (supplementary fig. S6, Supplementary Material online). It has a relatively low density of SNPs, suggesting relatively recent emergence. Several genes have the potential to be sex-determining. Bone morphogenetic protein and activin membrane–bound inhibitor (*bambi*) is a member of the TGF-β superfamily able to silence TGF-β signaling (Onichtchouk et al. 1999) which is involved in the regulation of ovarian follicle growth and maturation in mammals (Zhang et al. 2023). Mitogen-activated protein kinase 8 (*map3k8*) is expressed in the corpus luteum of mice and mediates the signaling pathway of estradiol (Liu et al. 2015). Zinc finger E-box binding homeobox 1 (*zeb1*) has been implicated in cellular proliferation and embryonic development (Lim and Song 2015). Bone morphogenetic protein 6 (*bmp6*) has been implicated in the regulation of AMH (Wang et al. 2023) and is involved in similar pathways to *bambi*. While none of these genes fall in the region with the highest sex-specific SNP density, which has no genes annotated on the *M. zebra* reference, they are strong candidates for loci affecting sex determination.

### Pseudocrenilabrus nicholsi

**Fig. 4.**
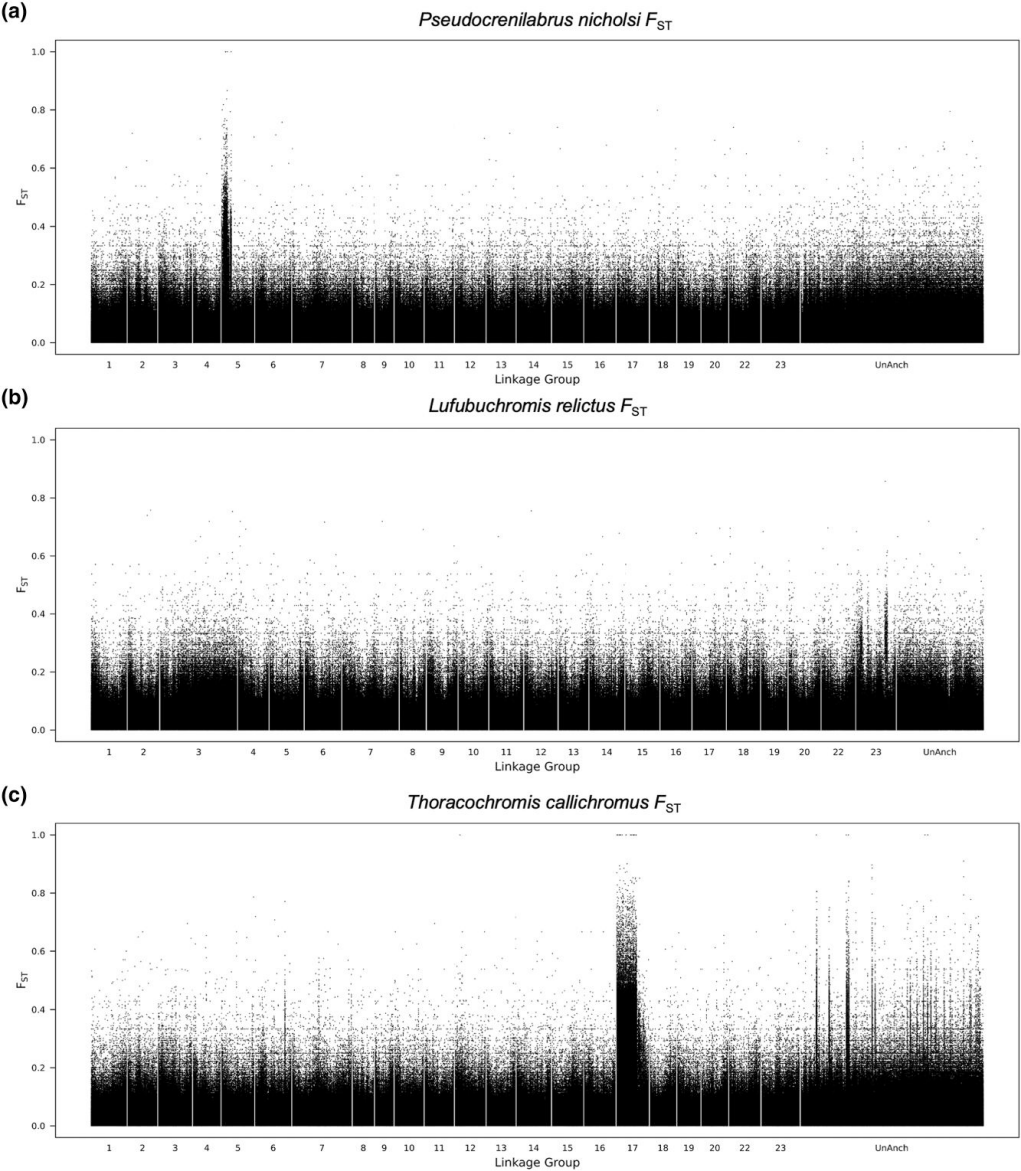
Whole-genome *F*_ST_ plots on the *M. zebra* UMD2a reference for a) *P. nicholsi*, b) *L. relictus*, and c) *T. callichromus*.

The *F*_ST_ plot shows strong signals on LG5 (Fig. 4a). Curiously, the detail plot for LG5 (supplementary fig. S7, Supplementary Material online) shows XY signal from 2 to 6 Mb and ZW signal from 9 to 11 Mb. Among the top 1% blocks of sex differentiation, there are 43 XY and 17 ZW blocks on LG5. Within the highest window of XY differentiation (5.7 Mb) is contactin4 (*cntn4*), a cell adhesion molecule with no obvious relationship to sex determination. Within the highest window of ZW differentiation (9.9 Mb) is *mgai1*, encoding a membrane-associated guanylate kinase which has been implicated in apoptosis of granulosa cells (Yu et al. 2017).

There is also XY signal on LG2 from 19 to 22 Mb (supplementary fig. S7 and table S7, Supplementary Material online). This region is supported by 15 XY blocks among the top 1% of 100 kb windows. There are approximately 20 sex-patterned SNPs per 100 kb window in this differentiated block, suggesting a second sex chromosome segregating in this population. The most differentiated XY window on XYLG2 (20.6 Mb) is fms-related receptor tyrosine kinase 4 (*flt4*), a receptor for vascular endothelial growth factors.

We are mystified by the conflicting XY and ZW signals on LG5. We considered the possibility that we see signal for a ZW system because of a large deletion on the Y that would make the XY male appear homozygous ZZ. We looked for differences in coverage across this region and found none that would suggest this is the case (supplementary fig. S7f, Supplementary Material online). The average number of sex-specific SNPs per 100 kb is 127 in the ZW region and only 70 in the XY region, suggesting that the ZW region could be older. However, even if a Y arose on an ancient W, it would not result in this pattern. We hypothesize that there are both XY and ZW systems segregating on LG5, with an additional XY locus on LG2. Without further genotyping, it is not possible to tease apart what epistatic relationships might exist among these three chromosomes.

### L. relictus

The *F*_ST_ plot shows three peaks of differentiation on LG23 (Fig. 4b). The three peaks do not coalesce when the analysis is performed on the *O. niloticus* assembly (supplementary fig. S8, Supplementary Material online) suggesting that there have been structural rearrangements in the *Lufubuchromis* lineage. The analysis of top 1% windows identifies 46 XY windows on LG23. The plot of XY-patterned SNPs also suggests XY signal on LG9, but this is not supported by the top 1% windows which include only one XY and one ZW window on LG9 (supplementary table S8, Supplementary Material online). There are approximately 20 sex-patterned SNPs per 100 kb window in the differentiated regions on LG23, suggesting a relatively recent origin of this sex chromosome. *Amh* is within the second block of differentiation at 10.1 Mb, but other regions show greater differentiation. No structural or coverage changes that would support *amh* as the sex determiner were noted.

### T. callichromus

The *F*_ST_ plot shows a strong signal over the first 22 Mb of LG17 (Fig. 4c and supplementary fig. S9, Supplementary Material online). All 79 of the top 1% blocks are XY blocks on LG17 (supplementary table S9, Supplementary Material online). The peaks observed on the unanchored contigs of the *M. zebra* assembly coalesce when the data are plotted on the *O. niloticus* reference (supplementary fig. S9, Supplementary Material online). There are about 500 sex-patterned SNPs per 100 kb window, suggesting a relatively old sex chromosome, and no obvious evolutionary strata within the differentiated region.

### Similarities Among Syntenic Systems

The differentiation in the XY system on LG7 in *P. philander* from Thamalakane River overlaps the previously described XY system on LG7 in *P. philander* from Lake Chila (Böhne et al. 2019). These two populations are over 1,500 km apart, raising the question of whether they had a shared origin. Comparison of the male-specific *k*-mers on LG7 from each population identified 161,040 shared *k*-mers on LG7, indicating a shared origin of this sex-determining system. The distribution of these *k*-mers across LG7 reflects the SNP density pattern seen in each of the respective systems separately (supplementary fig. S10, Supplementary Material online). This elevated number of shared *k*-mers on LG7 was not seen in comparisons with *P. philander* from Lake Mweru (Chila × Mweru = 8,146 *k*-mers, Thamalakane × Mweru = 16,543), and in these comparisons, the shared *k*-mers were not concentrated in the sex-determining region.

### Turnover Rates

We estimated the rate of sex chromosome turnover in *Pseudocrenilabrus* + *Lufubuchromis* + *Thoracochromis* by dividing the number of turnovers (6) by the sum of the branch lengths (∼26 MY), yielding ∼0.23 turnovers per million years. However, in the seven populations of three nominal species of *Pseudocrenilabrus*, there were four turnovers over ∼10 MY of branch length, for a rate of ∼ 0.41 turnovers per million years.

### Distribution of New Sex Chromosomes

To estimate the number of autosomes that can become sex chromosomes in East African cichlids, we tabulated all of the published instances of sex chromosomes in East African cichlids (supplementary table S10, Supplementary Material online). We identified 51 independently evolved sex chromosome systems involving 20 of 22 chromosomes in the standard karyotype. In addition, we identified independent evolution of sex determiners on unpaired B chromosomes in Lakes Malawi and Victoria.

**Fig. 5.**
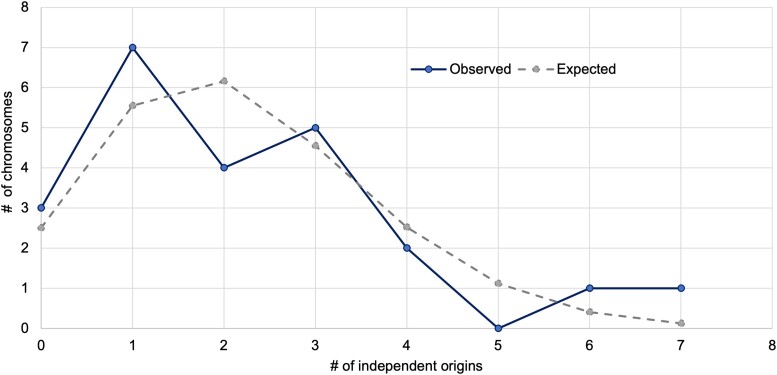
Distribution of independent origins of sex chromosome on 23 chromosomes in East African cichlids and best fit of the data to a Poisson distribution with mean of 2.2 (χ^2^ test, *P* = 0.246).

The data are consistent with Poisson expectations (χ^2^, *P* = 0.246; Fig. 5 and Table 1). Two chromosomes were used many times: LG5 was used seven times and LG7 was used six times. However, LG7 is nearly double the average size of the remaining chromosomes and is therefore a larger target. Monte Carlo simulation revealed that LG7 was not used more often than random expectations (Bonferroni-corrected *P* = 0.4889), but LG5 was used more often than expected (Bonferroni-corrected *P* = 0.0086). Also, many of these events are mapped to different regions of these chromosomes. The regions of differentiation in the ZW system of the Cyprichromini and the XY systems in *T. callichromus* and *A. burtoni* overlap the ZW system in Lake Malawi cichlids, but the XY systems on LG5 in the Tropheini do not. It remains unclear how many different genes are involved in the sex chromosome systems located on this chromosome.

**Table 1:** Number of independent recruitments of each LG as a sex chromosome in East African cichlids

LG	No. of occurrences
LG01	1
LG02	3
LG03	4
LG04	3
LG05	7
LG06	0
LG07	6
LG08	0
LG09	1
LG10	1
LG11	1
LG12	1
LG13	1
LG14	2
LG15	3
LG16	3
LG17	2
LG18	1
LG19	2
LG20	3
LG22	0
LG23	4
B	2
Sum	51

## Discussion

### Ancestral State of the Haplochromines

**Fig. 6.**
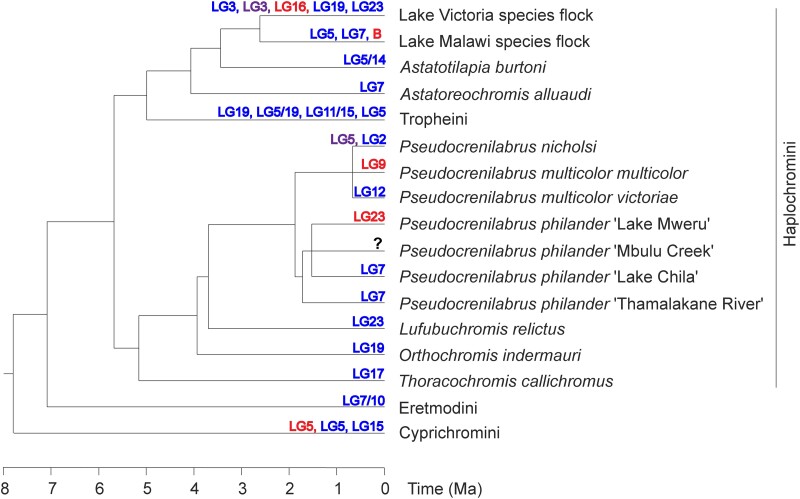
Sex chromosome turnovers in basal haplochromine cichlids in the context of adjacent groups. Topology is derived from Ronco et al. (2021) and Astudillo et al. (2023), with species not included in these studies incorporated according to their phylogenetic position and relative divergence times in other studies (Koblmüller, Schliewen, et al. 2008; Irisarri et al. 2018; Meier et al. 2019; Schedel et al. 2019). Blue indicates an XY system, red indicates a ZW system, and purple indicates the presence of both XY and ZW signal on the same chromosome.

One goal of this study was to identify the ancestral sex chromosome state for the “modern” haplochromines (sensu Salzburger et al. 2005) which includes the diverse radiations of cichlids in Lakes Malawi and Victoria (incl. the surrounding smaller rift lakes), as well as the radiation of Tropheini in Lake Tanganyika and some riverine species. *Pseudocrenilabrus* is an immediate sister group to the modern haplochromines, but the phylogeny for *Pseudocrenilabrus* is not completely resolved (Fig. 6). It is possible that *P. multicolor* is not monophyletic (Egger et al. 2015), and this uncertainty is represented by a polytomy in our phylogeny. Similar uncertainty exists for the relationship between the *P. philander* populations, which is also represented by a polytomy. The presence of two distinct mtDNA haplotypes in Lake Chila (Egger et al. 2015) suggests the possibility of a recent human-mediated migration which may have introduced the XY-LG7 system.

We considered two candidates for the ancestral sex chromosome state of the modern haplochromines. The XY-LG7 system is the most differentiated sex chromosome in *Pseudocrenilabrus* and is thus a good candidate for the ancestral state of the genus. XY-LG7 systems have been identified also in several haplochromines from Lake Malawi and the nearby Lake Masoko (Ser et al. 2010; Munby et al. 2021). However, these systems evolved independently from the XY-LG7 in *P. philander* (Böhne et al. 2019) and thus do not provide support for the idea that XY-LG7 was the ancestral state. On the other hand, previous work suggested that the XY-LG19 system in *O. indermauri* and the *Tropheus* species of Lake Tanganyika had a shared origin (El Taher et al. 2021). The evidence for this relied on a phylogenetic analysis of X and Y haplotypes inferred from short-read sequences. We attempted to confirm this through an analysis of shared *k*-mers, but the signal was not larger than expected by random convergence. Thus, we are not able to determine the ancestral state of the haplochromine ancestor with the data currently available.

### Rates of Sex Chromosome Turnover and Differentiation

The rate of sex chromosome turnover in cichlids is the highest known in vertebrates. Across the radiation of cichlids in Lake Tanganyika, it was estimated to be approximately 0.18 turnovers per million years (El Taher et al. 2021) and, for the tribe Tropheini, approximately 0.259 turnovers per million years (Behrens et al. 2024). Our estimate of the turnover rate in *Pseudocrenilabrus* + *Lufubuchromis* + *Thoracochromis* is similar, ∼0.23 turnovers per million years. In the seven populations of three nominal species of *Pseudocrenilabrus*, the estimate is slightly higher, ∼0.41 turnovers per million years. The actual rates could be higher depending on the ancestral state for the clades, or if there are unobserved turnovers, especially on the long branches leading to *Lufubuchromis* and *Thoracochromis*.

Quantifying the relative age of sex chromosomes is challenging; however, we can get an approximate estimate of levels of differentiation using sex-specific SNP density. The sex-specific SNP density and *F*_ST_ statistics are compromised by the frequency of a system in the population, so low-frequency systems will be more difficult to detect and will appear to have fewer sex-specific SNPs. In these basal haplochromines, SNP density is consistent with the proposed recent turnovers. The maximum density of SNPs in a 100 kb window on LG7 in *P. philander* “Thamalakane River” (700 SNPs; supplementary fig. S1, Supplementary Material online) and *P. philander* “Lake Chila” (450 SNPs; supplementary fig. S2, Supplementary Material online) corroborates the *k*-mer data indicating a relatively old but shared origin of this system. The high sex-specific SNP density on LG17 in *T. callichromus*, which also has a maximum of 700 SNPs per 100 kb window (supplementary fig. S9, Supplementary Material online), also suggests a relatively old system. In contrast, systems that may have experienced more recent turnover, such as *P. philander* “Lake Mweru” (25 SNPs; supplementary fig. S4, Supplementary Material online) and *P. multicolor victoriae* (150 SNPs; supplementary fig. S5, Supplementary Material online), have much lower maximum SNP densities in their sex-determining regions. Our estimated sex turnover rate for basal haplochromines may be an underestimate because we have likely missed intermediate sex chromosome systems. Estimates of the rates of sex chromosome turnover in cichlids will probably continue to increase as more species are investigated.

### Unlimited Options?

Our data on the chromosomal locations of 51 independent origins of sex chromosome systems in cichlids closely matches Poisson expectations. All but two cichlid chromosomes have become sex chromosomes in one lineage or another. Only one chromosome (LG5) has become a sex chromosome more often than random expectation. There seems little reason to believe that only a few chromosomes can take on the role of sex determiner in this group.

Because our data only maps the location of the sex determiner, it is possible that these diverse sex chromosome systems are the result of one or a few sex-determining genes duplicating or transposing to new locations, as observed in strawberries (Tennessen et al. 2018), pufferfish (Kabir et al. 2022), and salmonids (Yano et al. 2013). We think this is unlikely to be the case. If the same sequence was a sex determiner in each species, at least some sex-linked variation should be observed at a common location in the common reference genome assembly. However, transposition of a short or repetitive sequence controlling sex determination might not have been detected in our data. Clearly, further work to identify the causative variation in each lineage is required. To that end, we are actively sequencing male and female genomes from many of these cichlid lineages to enumerate the gene content within each new sex-determining region.

## Conclusion

This investigation has revealed an extraordinary diversity and high turnover rate of sex chromosomes in some basal haplochromines, exceeding previous estimates of sex chromosome turnover in cichlids. In *Pseudocrenilabrus*, we identified a relatively old sex chromosomes even while adjacent lineages have undergone comparatively recent sex chromosome turnover. Our statistical analysis of sex chromosomes in African cichlids suggests that each cichlid autosome has a roughly equal chance of becoming a sex chromosome, challenging the idea that only a handful of genes can be recruited to become master regulators of sex determination in vertebrates.

## Materials and Methods

### DNA Samples and Sequencing

We studied representatives of seven populations, including two subspecies of three described species of *Pseudocrenilabrus*. We also included *L. relictus* (Schedel et al. 2020) as an additional early branching representative of the *Pseudocrenilabrus*-like clade and *T. callichromus* as a representative of the *Serranochromis*-like cichlids, the sister group of the *Pseudocrenilabrus*-like clade. We obtained whole-genome sequences from seven full-sib families obtained from the aquarium trade (parents likely F_2_) and raised at the University of Graz: *P. philander* (25M, 24F) from the Thamalakane River near Maun, Botswana; *P. philander* (22M, 30F) from Lake Mweru, Northern Zambia; *P. multicolor victoriae* (26M, 27F) from the Albert-Nile drainage; *P. multicolor multicolor* (13M, 22F) from the Nile River; *P. nicholsi* (17M, 22F) from the Lualaba River drainage, DR Congo; *L. relictus* (14M, 13F) from the upper Lufubu River, Zambia; and *T. callichromus* (25M, 21F) from Lake Fwa, Central DR Congo. We also reanalyzed previously published data consisting of whole-genome sequences of *P. philander* from Lake Chila (6M, 6F) and Mbulu Creek (4M, 3F) in Zambia (GenBank accession PRJNA472096) (Böhne et al. 2019).

Animal use was approved under animal care protocols BMWFW-66.007/004-WF/V/3b/2016 (University of Graz) and R-OCT-19-48 (University of Maryland). This study was carried out with the approval of the ethics committee of the University of Graz (permit number GZ. 39/115/63 ex 2022/23).

DNA was purified separately for each individual using phenol:chloroform extraction in phase-lock gel tubes (Quantabio, Beverly MA). The DNA from each individual was quantified by PicoGreen fluorescent assay (Thermo Fisher Scientific, Waltham, MA), and then, equimolar pools were constructed for males and for females. Sequencing libraries were constructed for 150 bp paired-end DNA sequencing on a NovaSeq6000 S4 (Illumina, San Diego, CA) by the Maryland Genomics Center (Baltimore, MD).

### Sex-Specific SNP Analysis

The main focus of our analyses is the identification and analysis of sex-specific SNPs. These SNPs were identified following our methods described previously (Behrens et al. 2022) using the sex–SNP–finder pipeline (Gammerdinger et al. 2018). Previously reported code from that study is available at https://github.com/Gammerdinger/sex-SNP-finder. Briefly, the sequence reads were aligned with BWA version 0.7.12 (Li and Durbin 2009) using the default parameters along with read group labels. We aligned all samples to the closest high-quality reference assembly, *Maylandia zebra* UMD2a (RefSeq GCF_000238955.4) (Conte et al. 2019). In some cases, some of the sex-specific signal mapped to unanchored contigs of the *M. zebra* assembly, so we remapped the reads to a more contiguous assembly, *O. niloticus* UMD_NMBU (RefSeq GCF_001858045.2) (Conte et al. 2019). At each variable nucleotide site, we calculated the *F*_ST_ statistic between the populations of male and female sequence reads. The resulting *F*_ST_ plots provide a first indication of the differentiation between male and female genomes. We further identified XY- and ZW-patterned SNPs as SNPs that were fixed (frequency less than 0.1) in one sex and polymorphic (frequency between 0.3 and 0.7) in the other sex. Separate plots of the allele frequency of XY- and ZW-patterned SNPs often allowed determination of the type of heterogametic system segregating (XY or ZW). Read mappings in candidate sex determination intervals were examined in the IGV (Thorvaldsdóttir et al. 2013) to look for evidence of sequence coverage differences, copy number variation, or other structural rearrangements.

We used Bedtools (Quinlan and Hall 2010) *make windows* and *coverage* to calculate the density of sex-patterned SNPs in 100 kb windows across the genome. We identified the top 1% of windows (∼78 of the 7,800 anchored windows) with the highest number of sex-patterned SNPs using the methodology described in Kocher et al. (2022). The log_2_(XY:ZW) ratio of SNP density was then calculated for each window. A KW test on the ranked data was conducted in R (v.2023.03.0 + 386) using kruskal.test from the *stats* package to determine if the log ratio differed among chromosomes. If the differences were statistically significant, the Dunn's test from the *rstatix* R package was conducted post hoc to determine which chromosomes differed significantly from one another with a Benjamini-Hochberg correction for multiple tests. Regions of elevated sex-specific SNP density were visualized in IGV to identify candidate sex–determining genes.

### *K*-mer Analysis

To identify sex-linked variants shared by species with the same sex chromosome system, we conducted a *k*-mer analysis. Following our previous work, we used Jellyfish v. 2.2.7 (Marcais and Kingsford 2011) to tabulate the frequency of *k*-mers of length 22 bases. Code from that study is available at https://github.com/KristenBehrens/K-mer-scripts. Briefly, for each shared sex chromosome system, a core region was identified using a python script that compares *k*-mers from each species and generates a list of *k*-mers shared by all species with that system. These *k*-mers were then aligned to the *M. zebra* reference genome (M_zebra_UMD2a) using BLAST (Camacho et al. 2009) to determine the core region of differentiation. SAMtools v. 1.10 (Danecek et al. 2021) was used for any necessary file format conversions, sorting, or indexing. This analysis was conducted to compare shared male-specific *k*-mers between *P. philander* Thamalakane River and *P. philander* Lake Chila (Böhne et al. 2019).

### Frequency Analysis

To fit a Poisson distribution to the distribution of novel sex chromosomes over chromosomes, we calculated the Poisson mean (51 events/23 chromosomes = 2.2 events/chromosome) and then calculated the expected Poisson distribution for this mean. We additionally simulated 1,000 replicates of samples of 51 chromosomes using the Monte Carlo method in R. The Monte Carlo sampling accounted for the difference in size between chromosomes, using *M. zebra* as the reference for chromosome size. Thus, we evaluated the distribution of recruitment of LG5 and LG7 to see if they were recruited more than expected with Bonferroni correction for multiple testing.

## Supplementary Material

evae152_Supplementary_Data

## Data Availability

Whole-genome sequencing reads from the pooled sequence of both sexes from the seven full-sib families have been deposited in the NCBI Short Reads Archive under BioProject accession number PRJNA1085211.
