# Supplementary material for: Diversity of Sex Chromosomes in Vertebrates: Six Novel Sex Chromosomes in Basal Haplochromines (Teleostei: Cichlidae)
Source: Genome Biol Evol. 2024 Jul 29;16(7):evae152. doi: 10.1093/gbe/evae152 (PMC11285159; doi:10.1093/gbe/evae152)
Supplement: evae152_Supplementary_Data [file evae152_supplementary_data.zip › Basal Haplochromines Supplemental Figures.docx]

**Supplemental Figures**

**
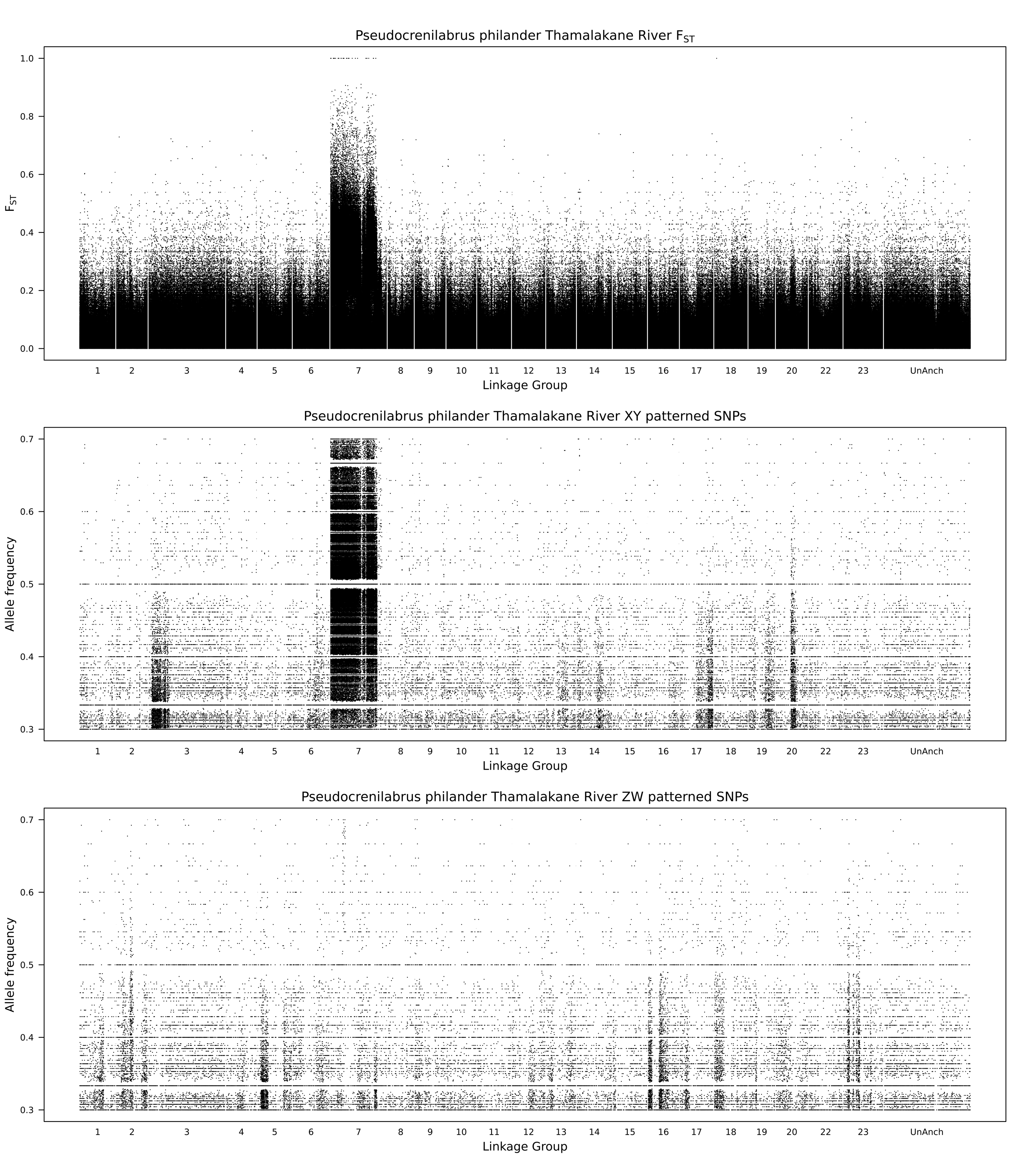
**

a

**
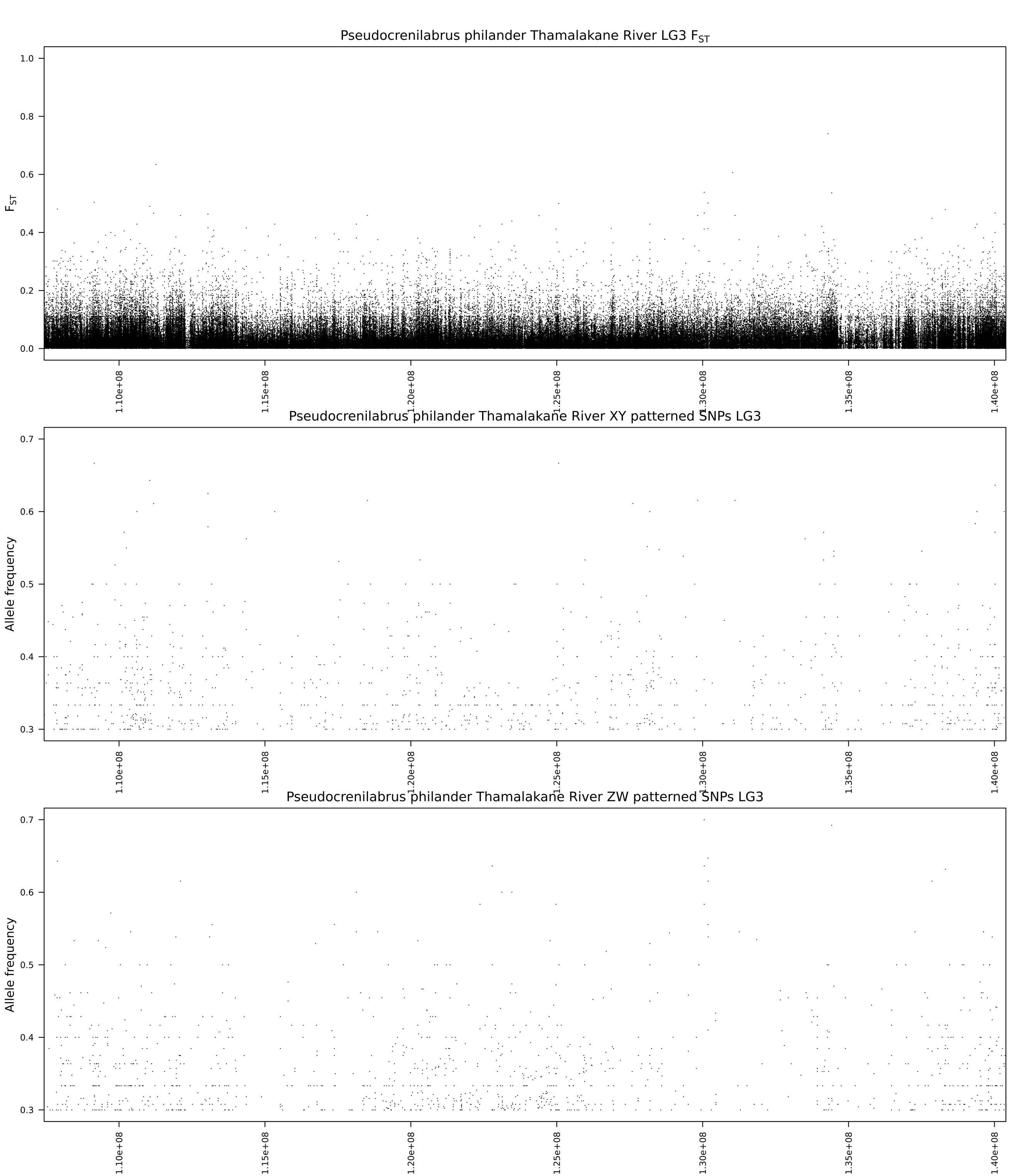
**

Position on chromosome (running genome size)

b

**
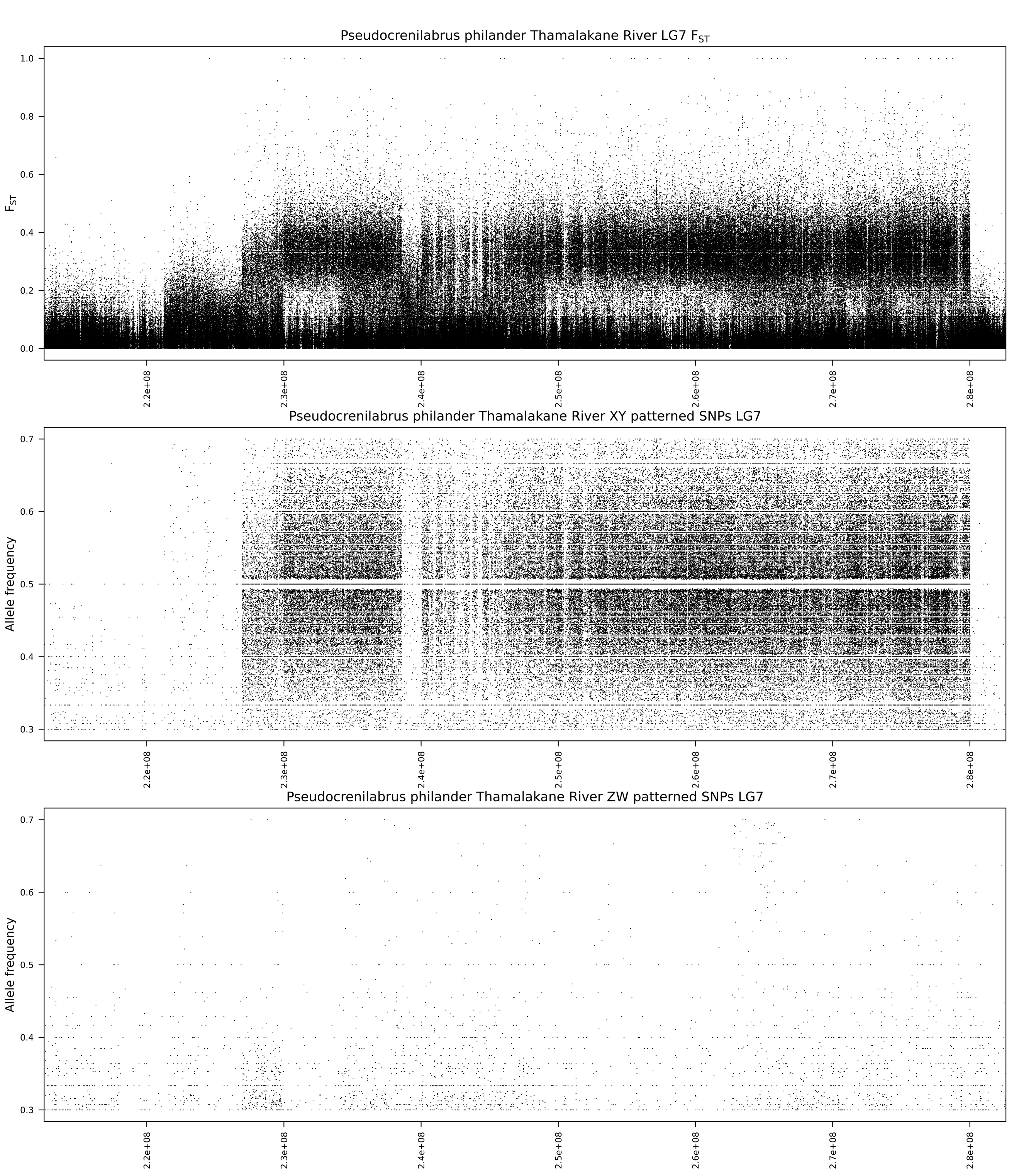
**

Position on chromosome (running genome size)

c

e

d

**Supplemental Figure 1.** *F*_ST_ and sex-patterned SNP plots for *Pseudocrenilabrus philander* (Thamalakane River), a) whole genome plot against *O. niloticus* reference, b,c) single chromosome *F*_ST_ and sex-patterned SNP plots against *M. zebra* reference, d,e) single chromosome sex-patterned SNP density per 100kb window plots against *M. zebra* reference


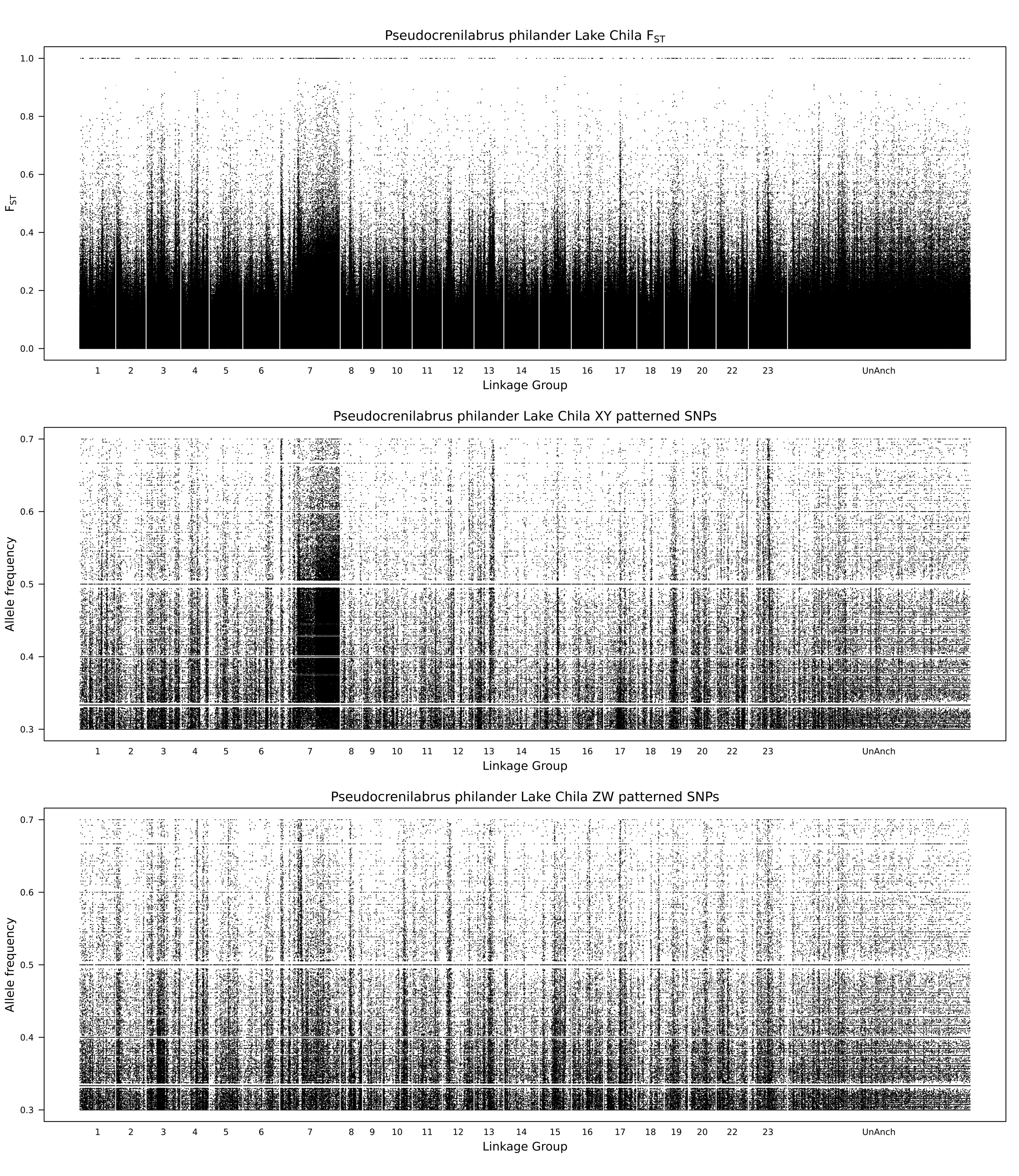


a


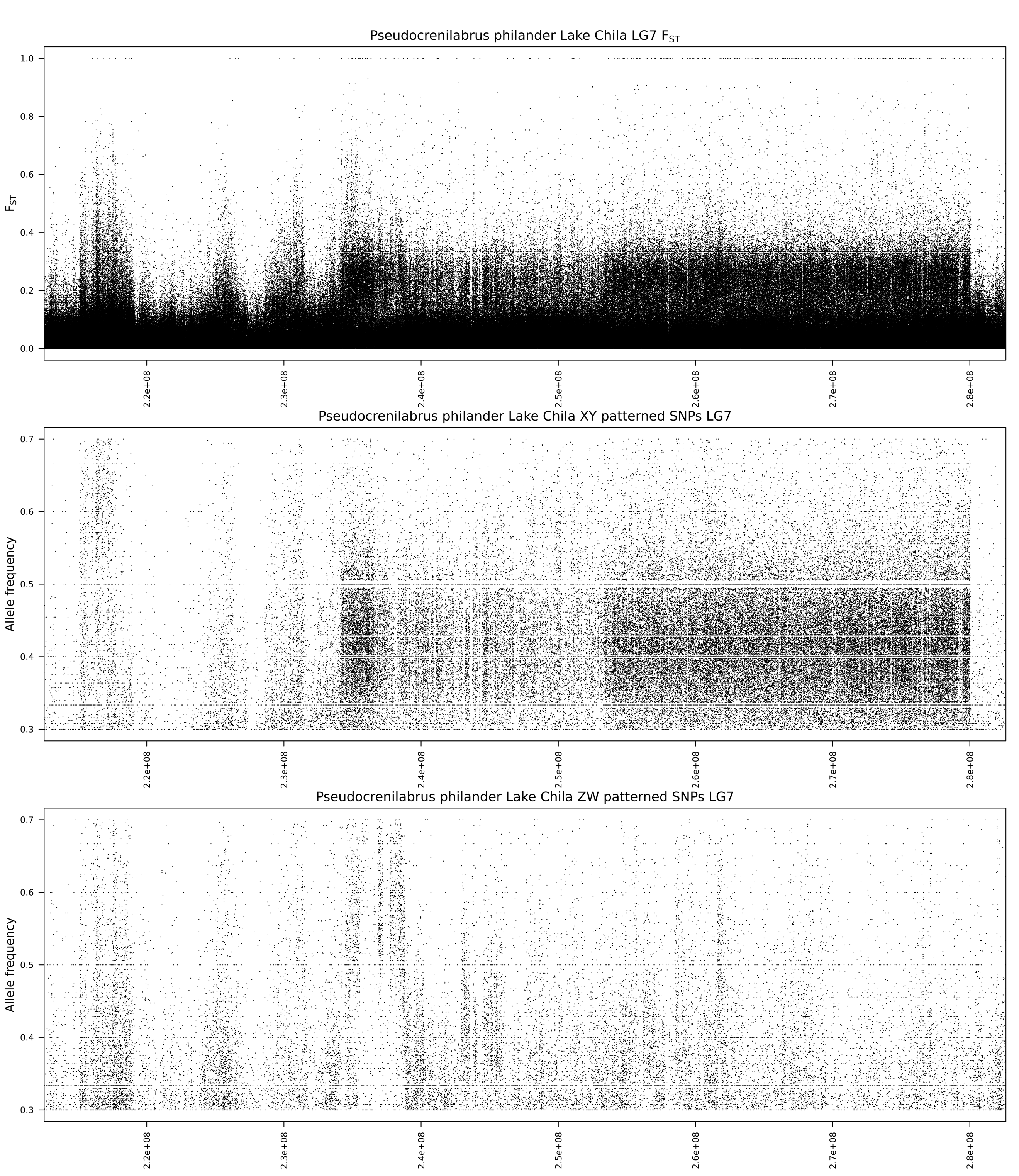


Position on chromosome (running genome size)

b

c

**Supplemental Figure 2**. *F*_ST_ and sex-patterned SNP plots for *Pseudocrenilabrus philander* (Lake Chila). a) whole genome plot against *M. zebra* reference, b) single chromosome *F*_ST_ and sex-patterned SNP plots against *M. zebra* reference, c) single chromosome sex-patterned SNP density per 100kb window plots against *M. zebra* reference


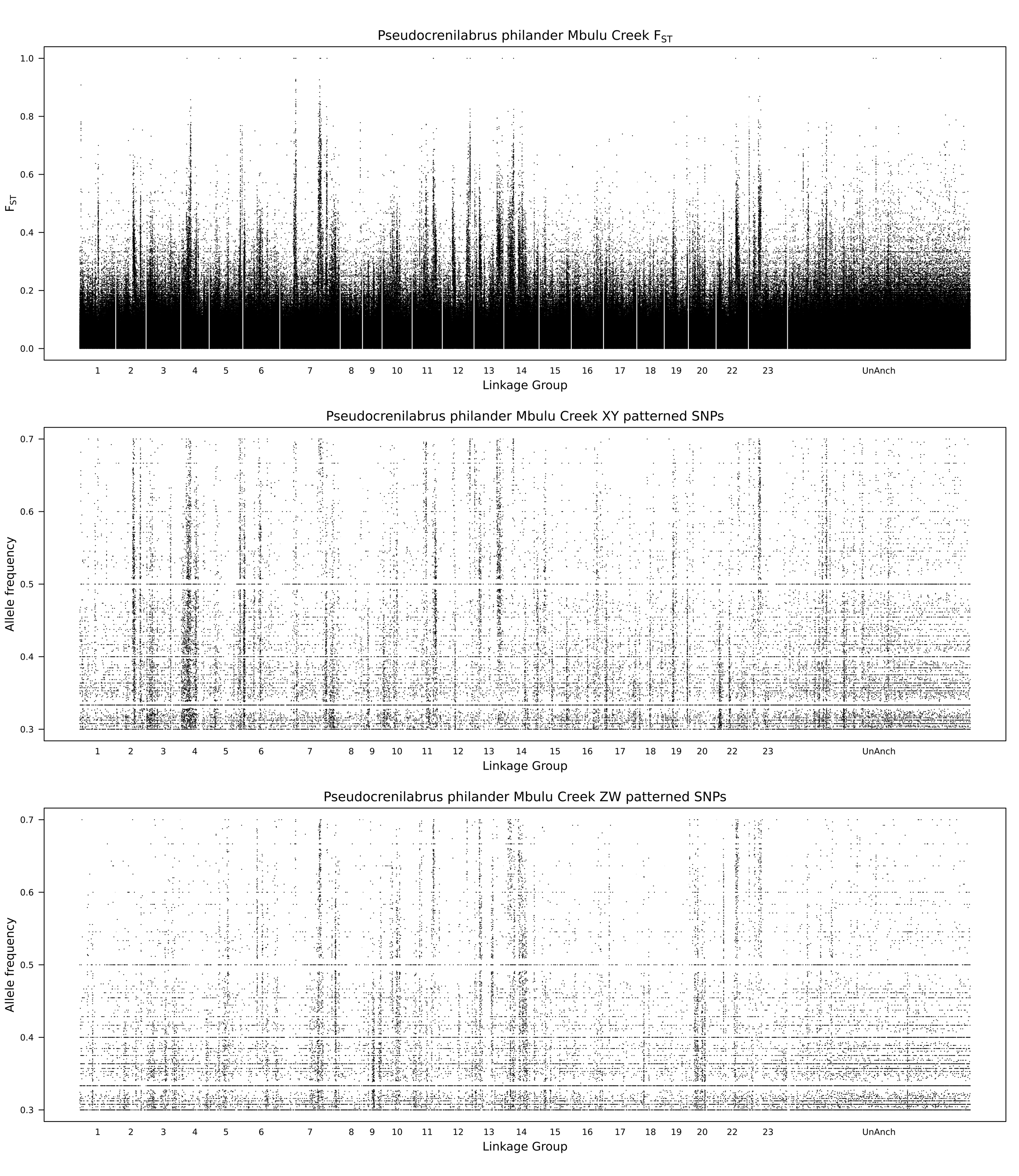


**Supplemental Figure 3.** *F*_ST_ and sex-patterned SNP plots for *Pseudocrenilabrus philander* (Mbulu Creek against *M.zebra* reference


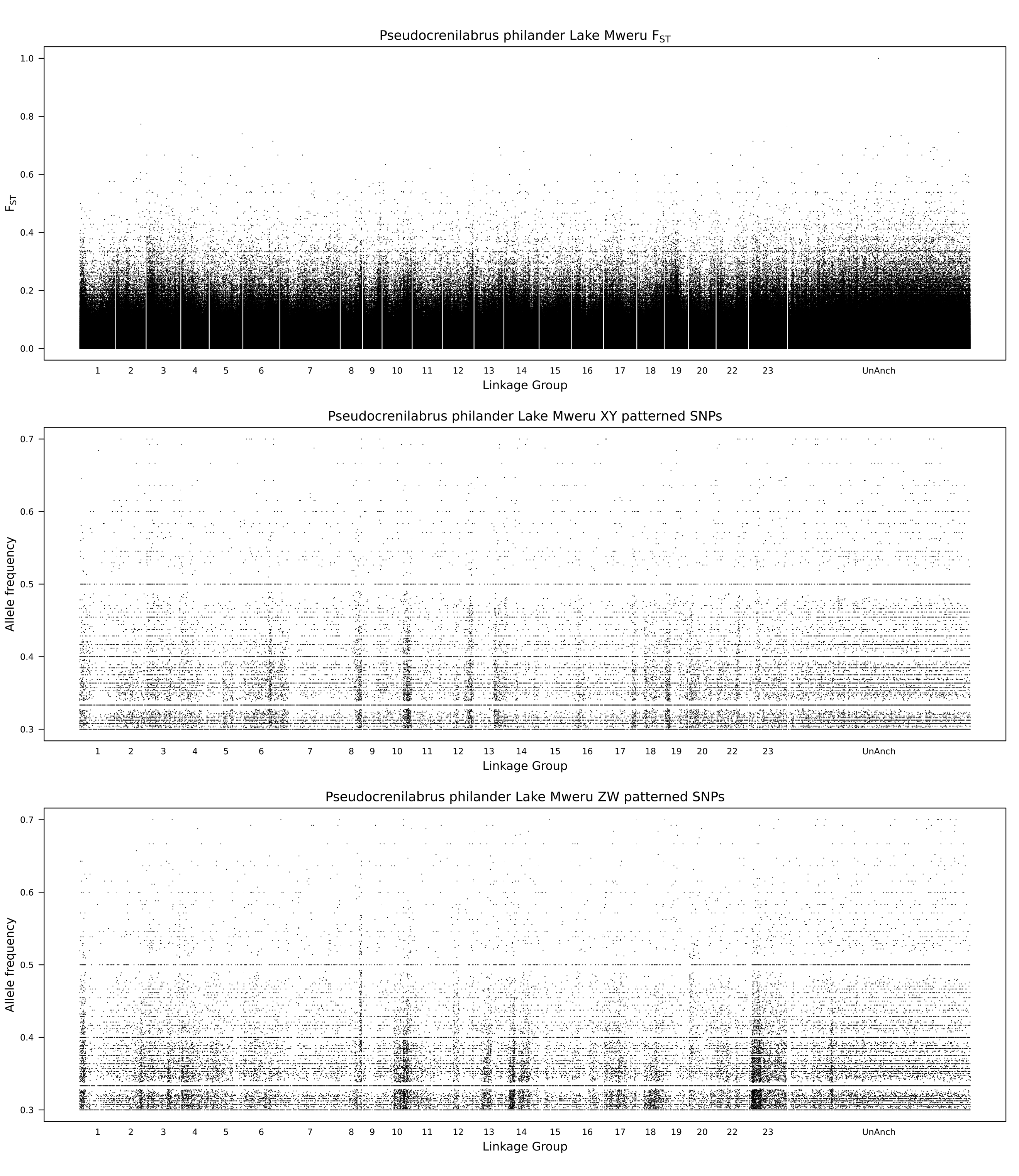


a

b

**Supplemental Figure 4.** *F*_ST_ and sex-patterned SNP plots for *Pseudocrenilabrus philander* (Lake Mweru). a) whole genome plot against *M. zebra* reference, b) single chromosome sex-patterned SNP density per 100kb window plots against *M. zebra* reference


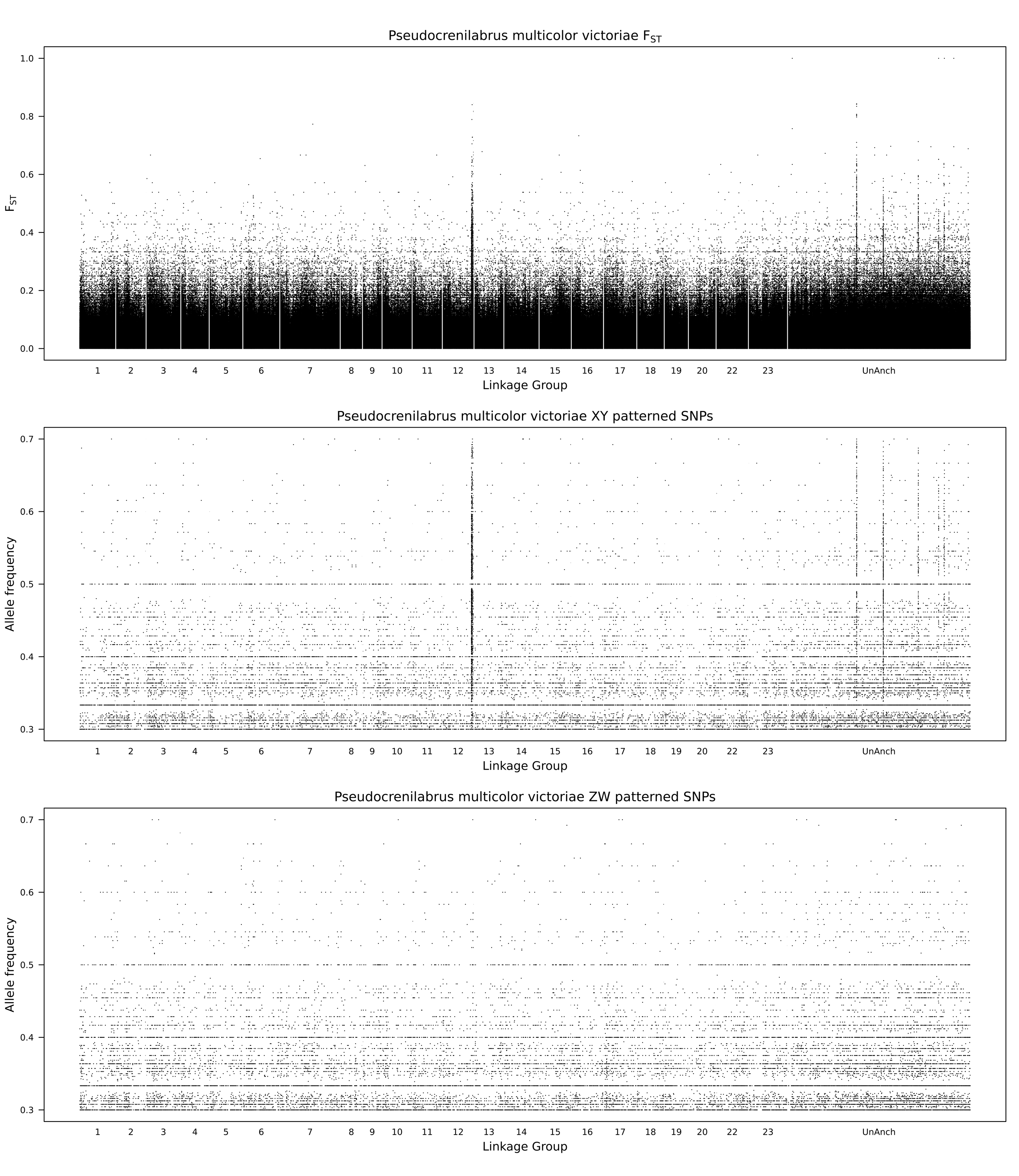


a


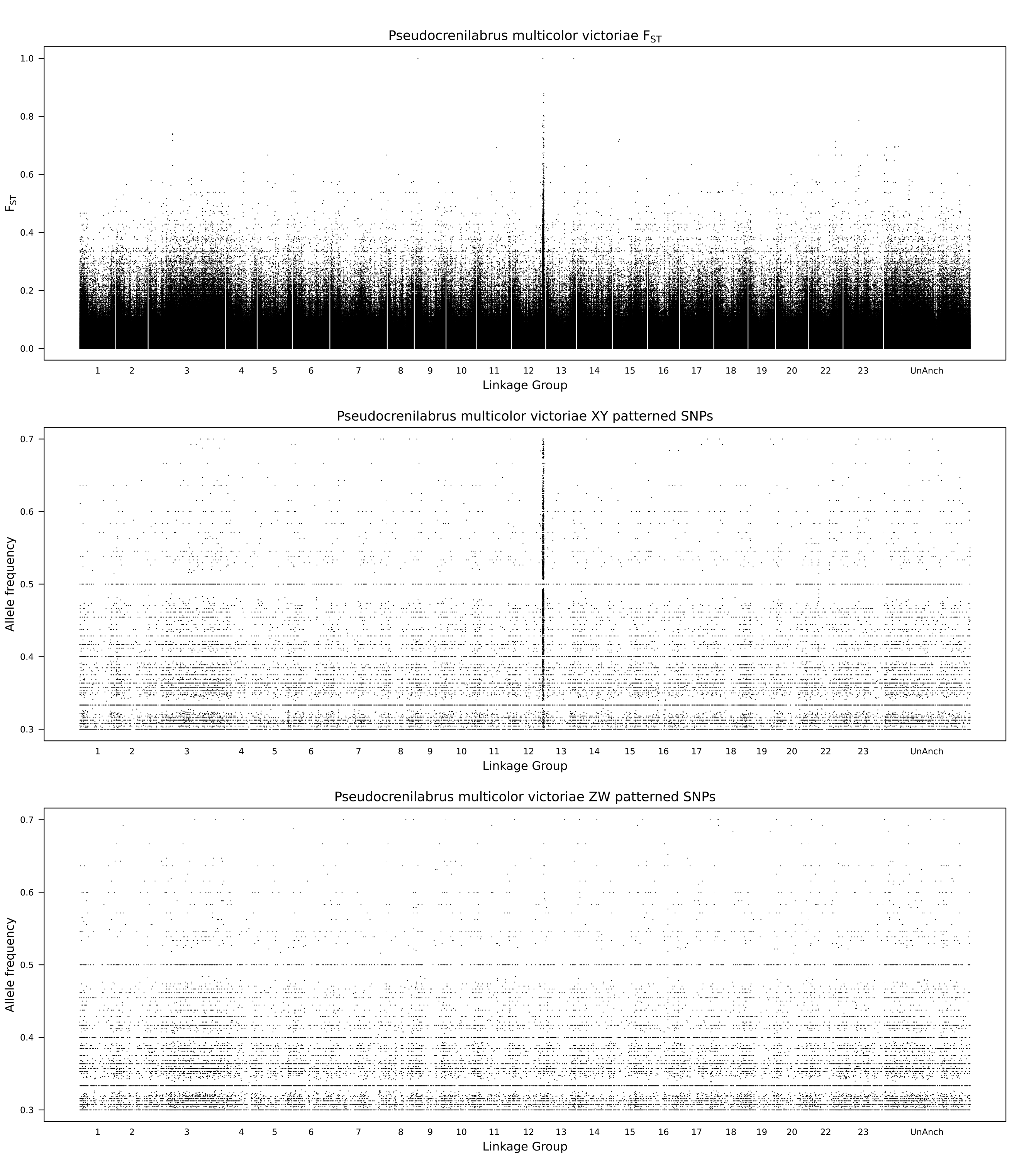


b


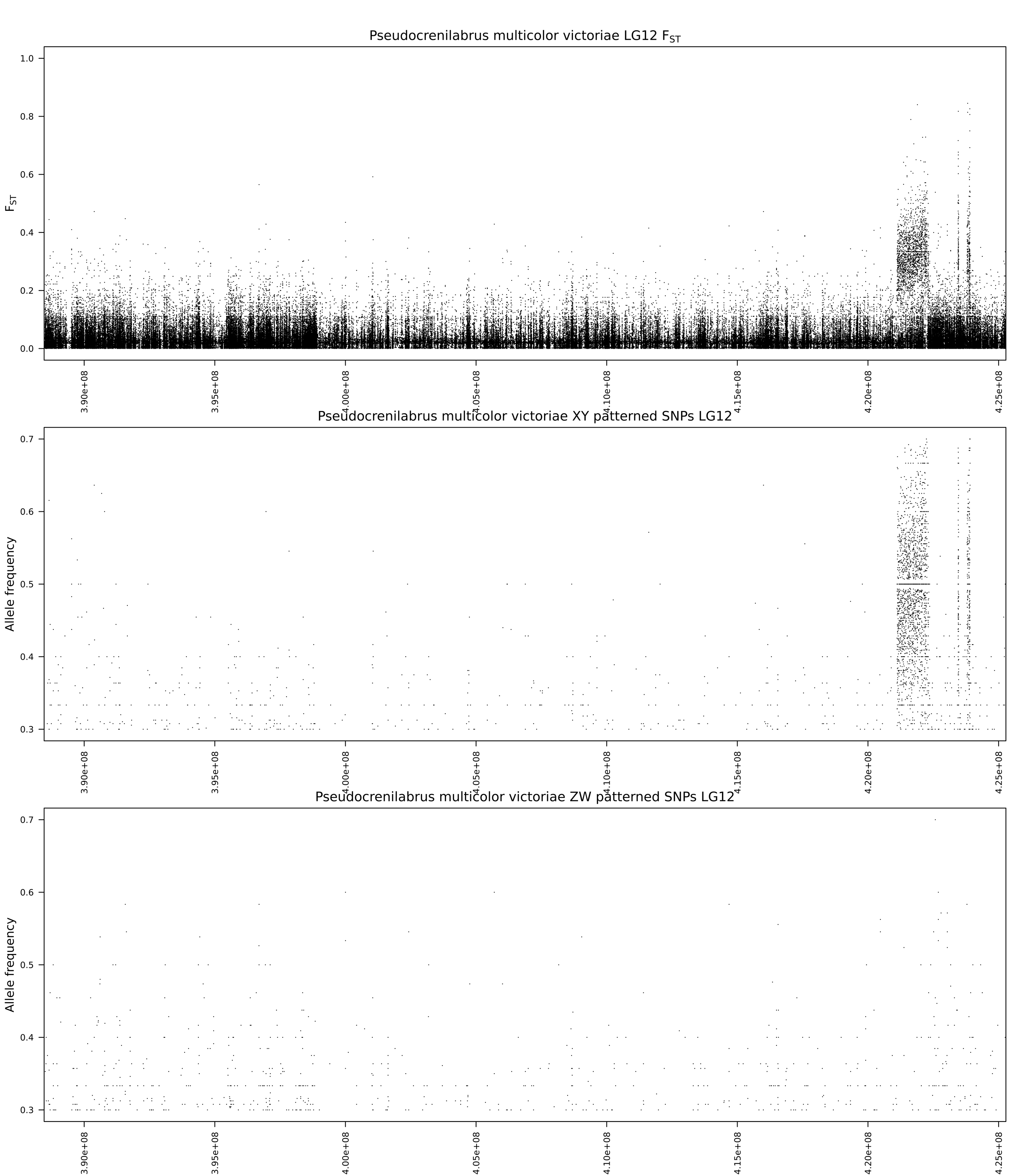


Position on chromosome (running genome size)

c

d

**Supplemental Figure 5.** *F*_ST_ and sex-patterned SNP plots for *Pseudocrenilabrus multicolor victoriae,* a) whole genome plot against *M. zebra* reference, b) whole genome plot against *O. niloticus* reference, c) single chromosome *F*_ST_ and sex-patterned SNP plots against *M. zebra* reference, d) single chromosome sex-patterned SNP density per 100kb window plots against *M. zebra* reference


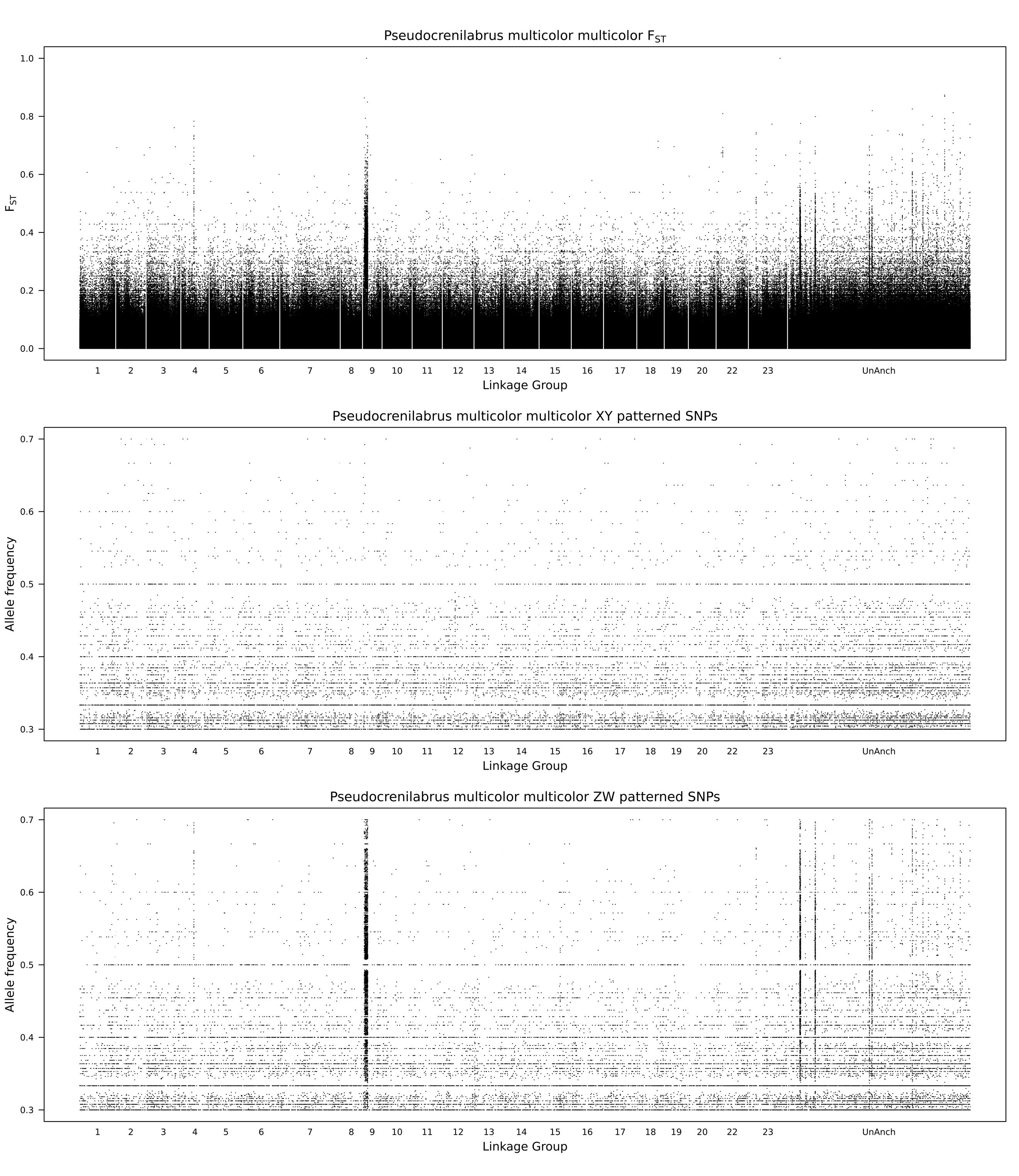


a


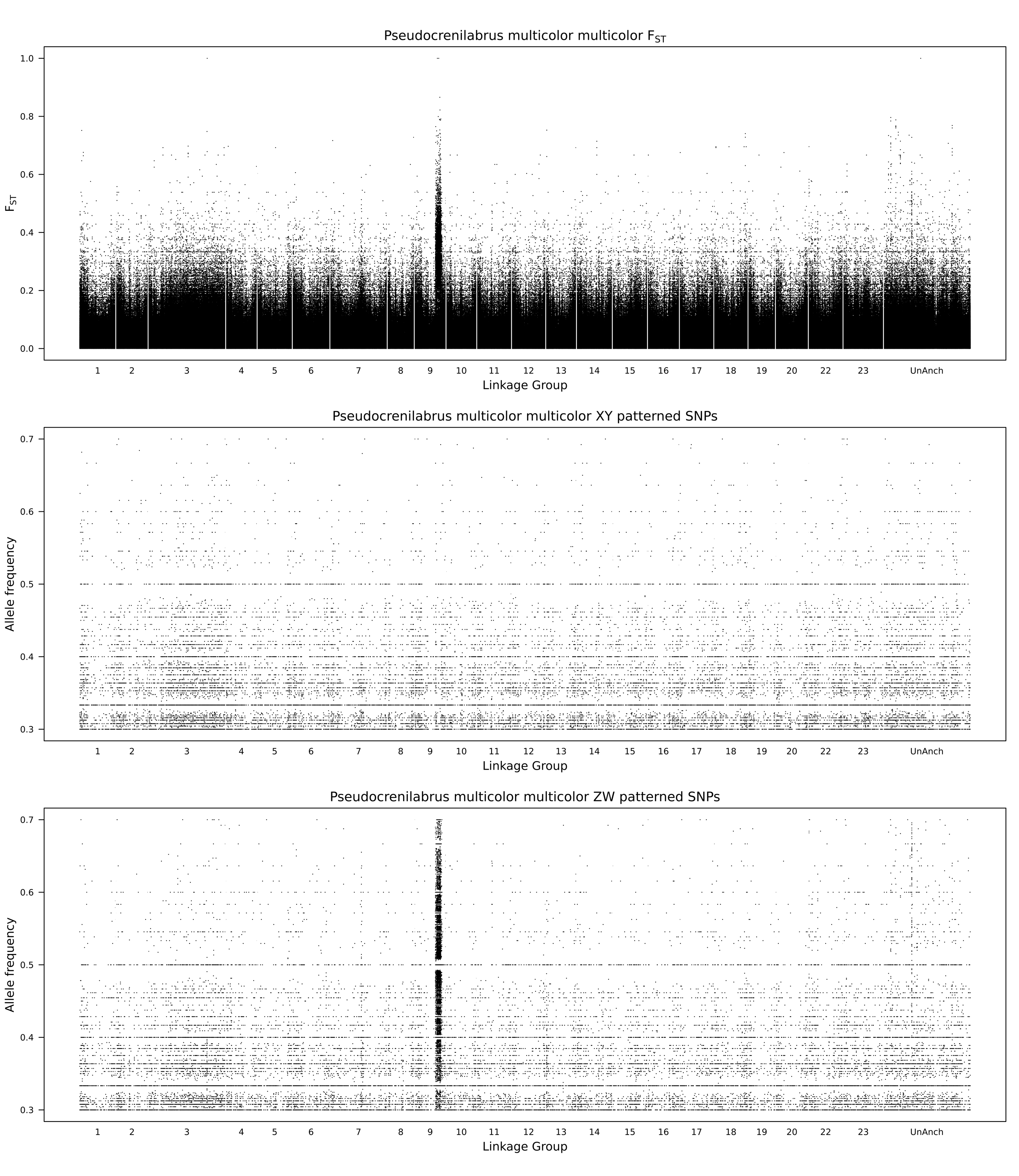


b


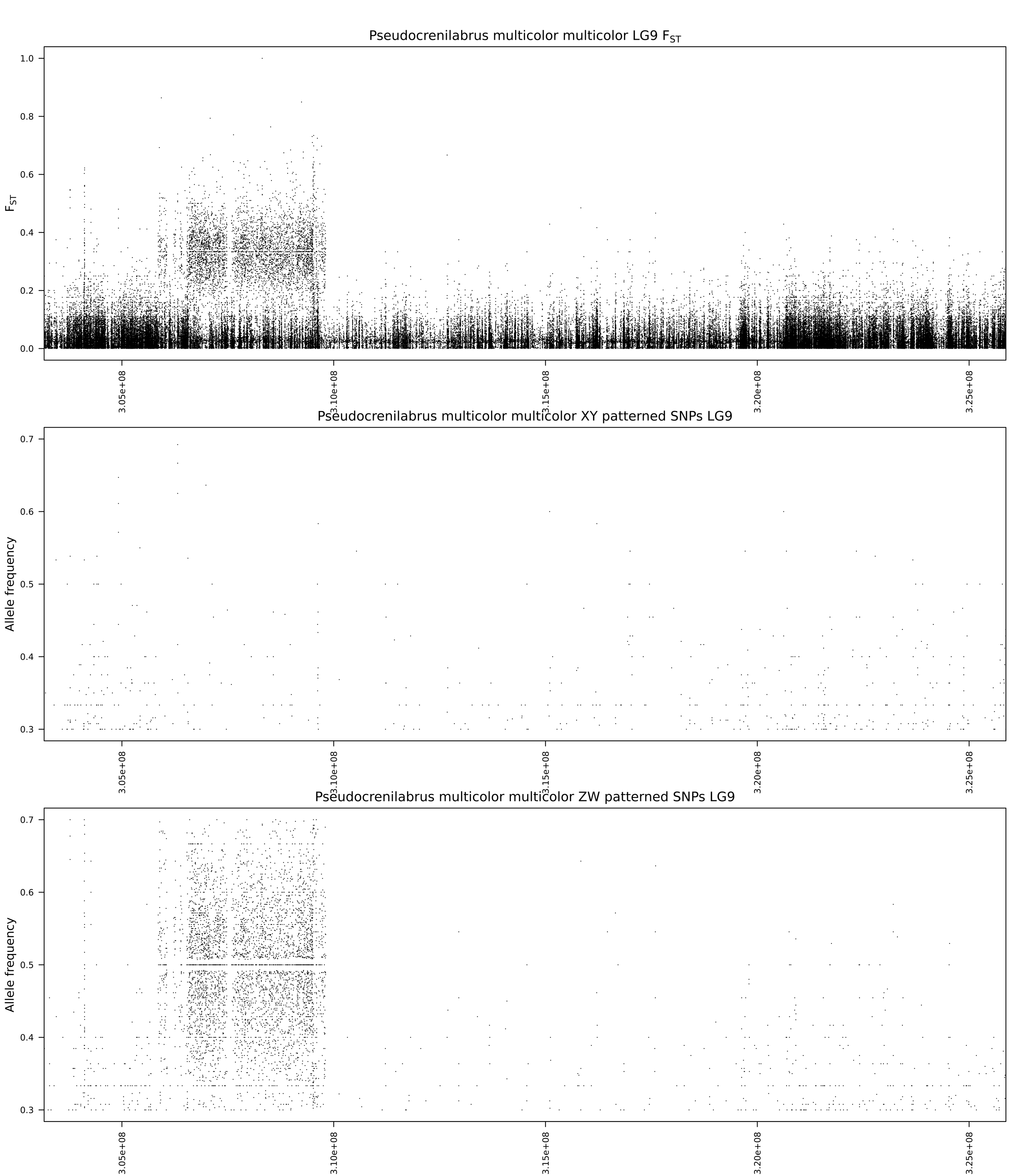


Position on chromosome (running genome size)

c

d

**Supplemental Figure 6.** *F*_ST_ and sex-patterned SNP plots for *Pseudocrenilabrus multicolor multicolor.* a) whole genome plot against *M. zebra* reference, b) whole genome plot against *O. niloticus* reference, c) single chromosome *F*_ST_ and sex-patterned SNP plots against *M. zebra* reference, d) single chromosome sex-patterned SNP density per 100kb window plots against *M. zebra* reference


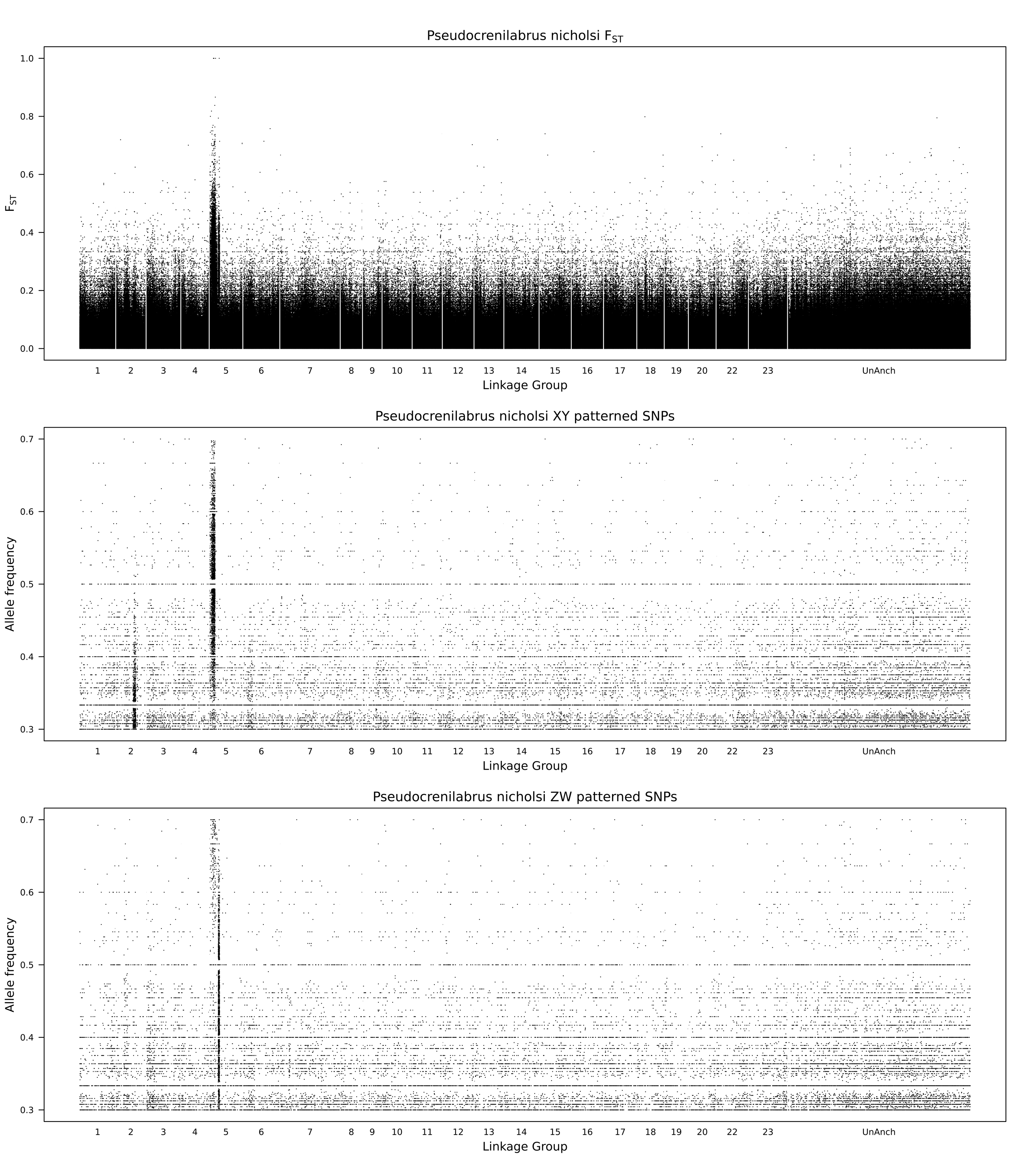


a


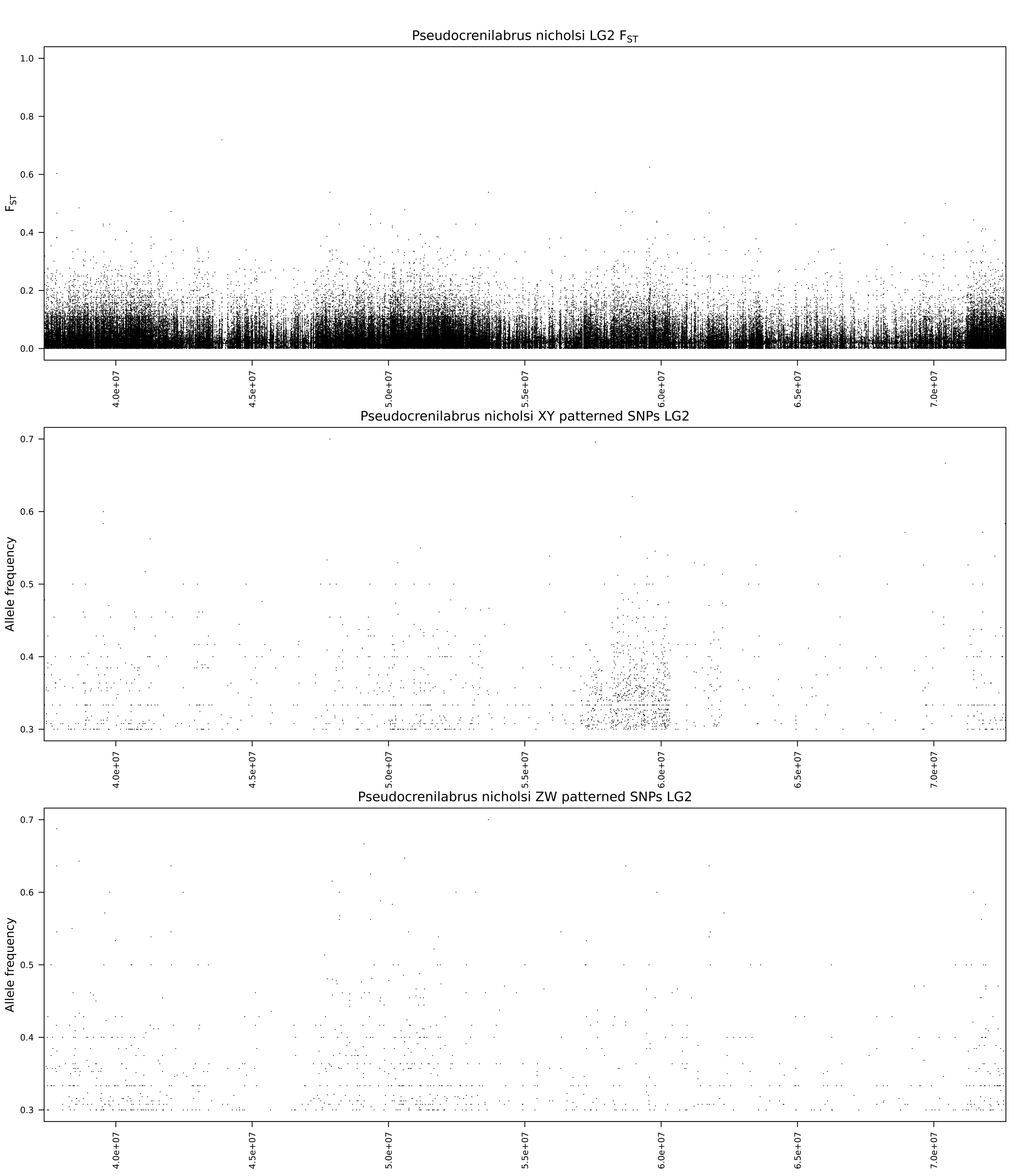


Position on chromosome (running genome size)

b


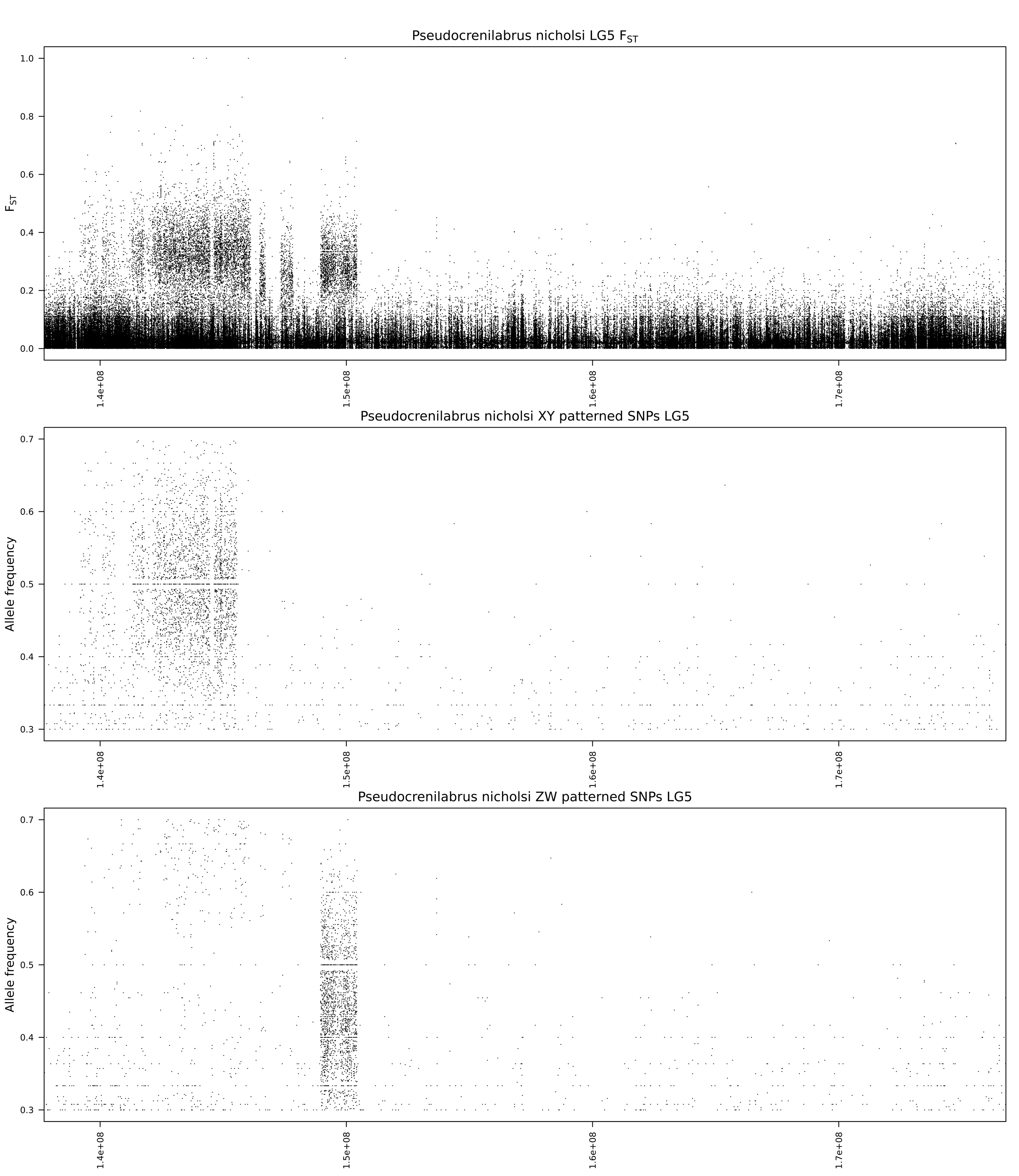


c

e

d

f

**Supplemental Figure 7.** *F*_ST_ and sex-patterned SNP plots for *Pseudocrenilabrus nicholsi.* a) whole genome plot against *M. zebra* reference, b,c) single chromosome *F*_ST_ and sex-patterned SNP plots against *M. zebra* reference, d,e) single chromosome sex-patterned SNP density per 100kb window plots against *M. zebra* reference f) log_2_(XY-ZW) of SNPs per 100kb window on LG5.

**
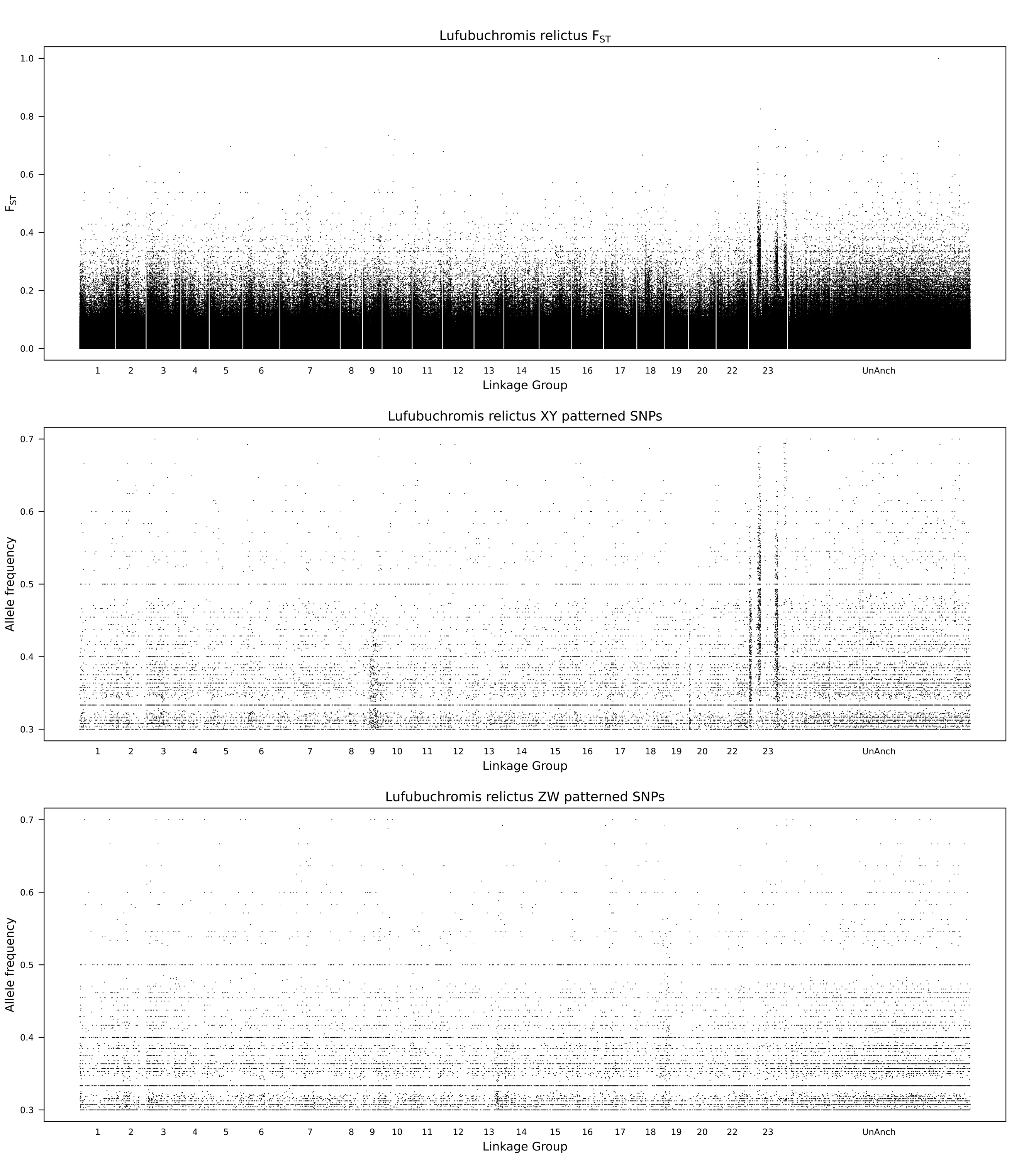
**

a

**
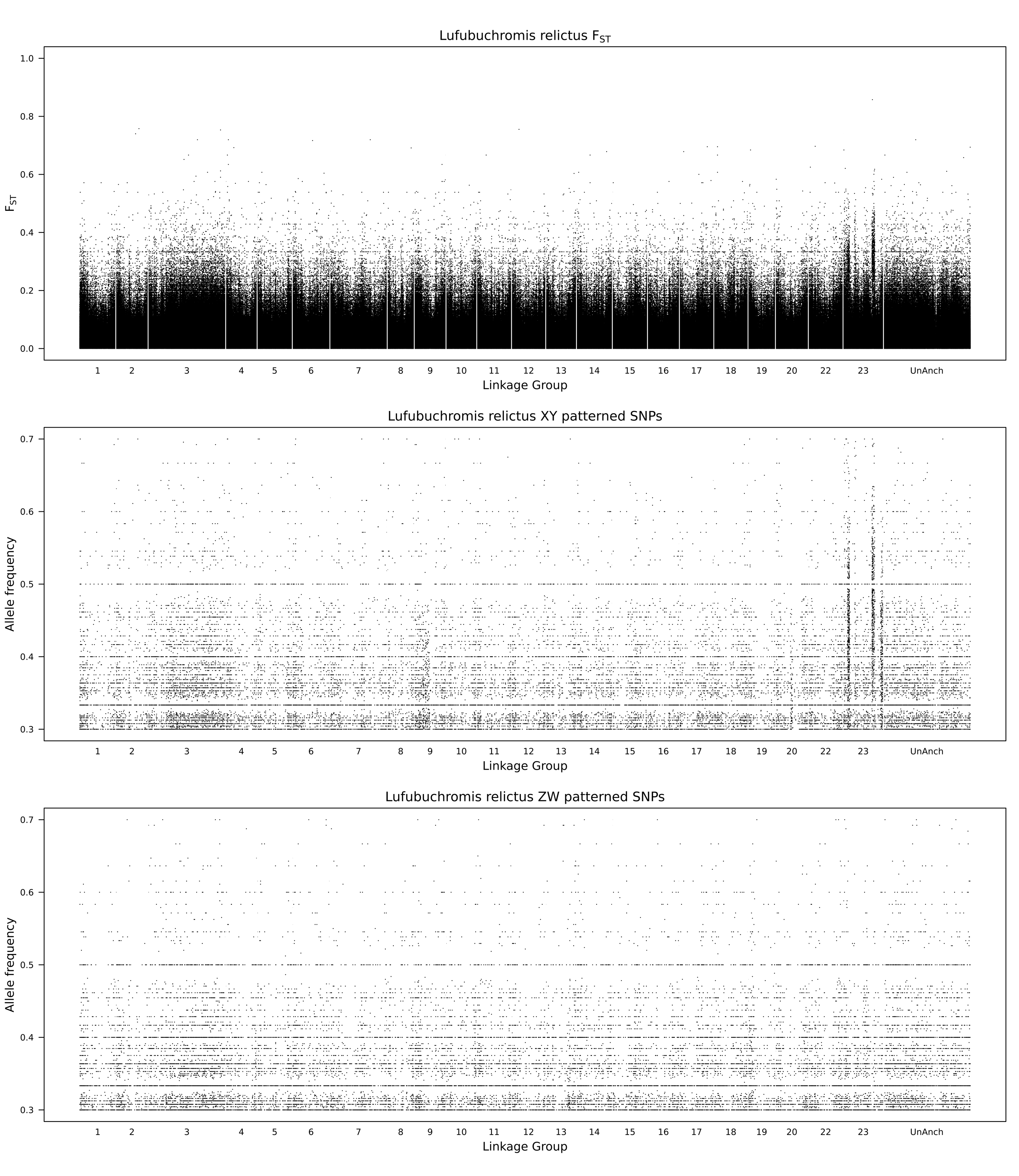
**

b

**
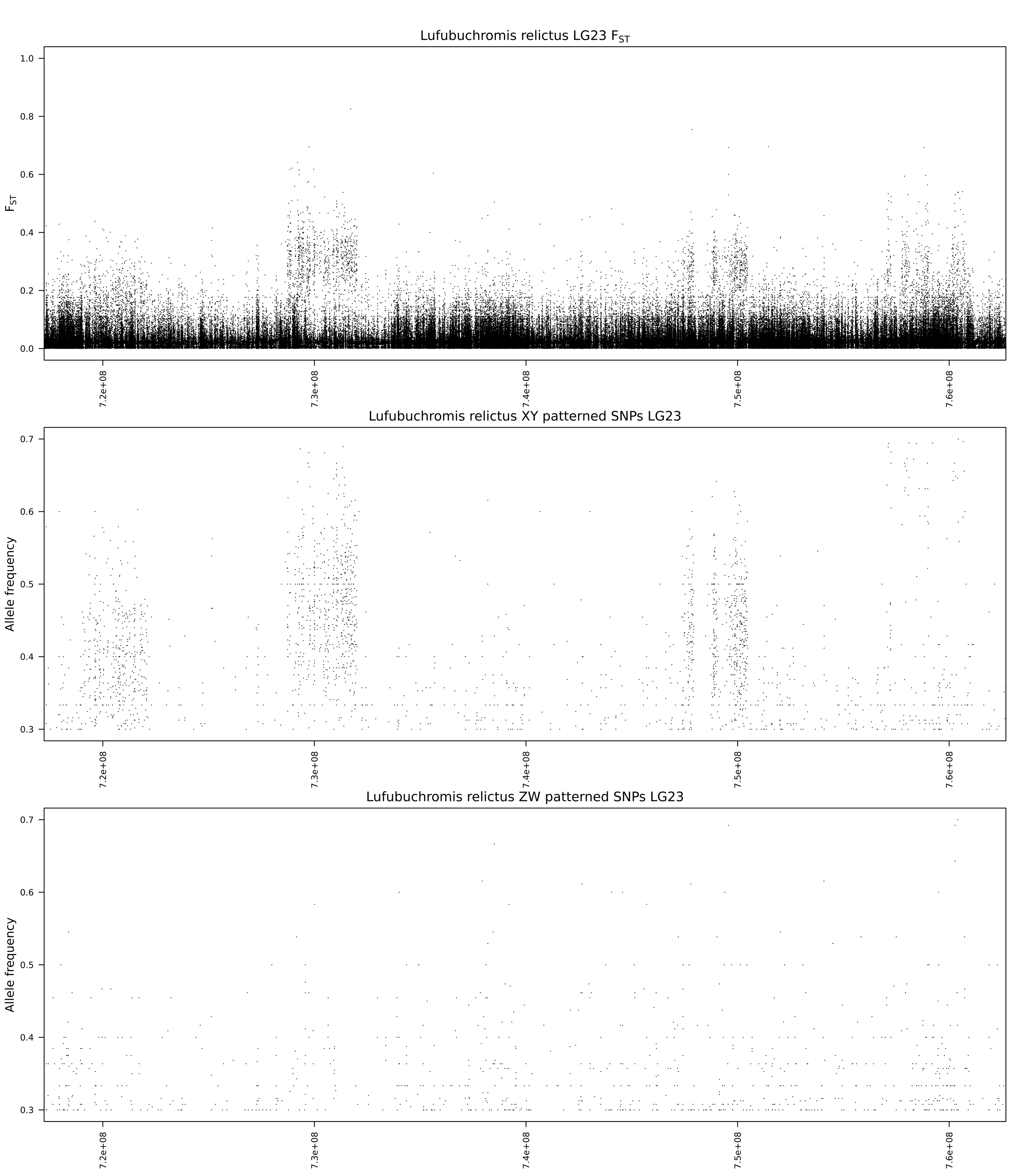
**

Position on chromosome (running genome size)

c

d

**Supplemental Figure 8.** *F*_ST_ and sex-patterned SNP plots for *Lufubuchromis relictus*. a) whole genome plot against *M. zebra* reference, b) whole genome plot against *O. niloticus* reference, c) single chromosome *F*_ST_ and sex-patterned SNP plots against *M. zebra* reference, d) single chromosome sex-patterned SNP density per 100kb window plots against *M. zebra* reference

**
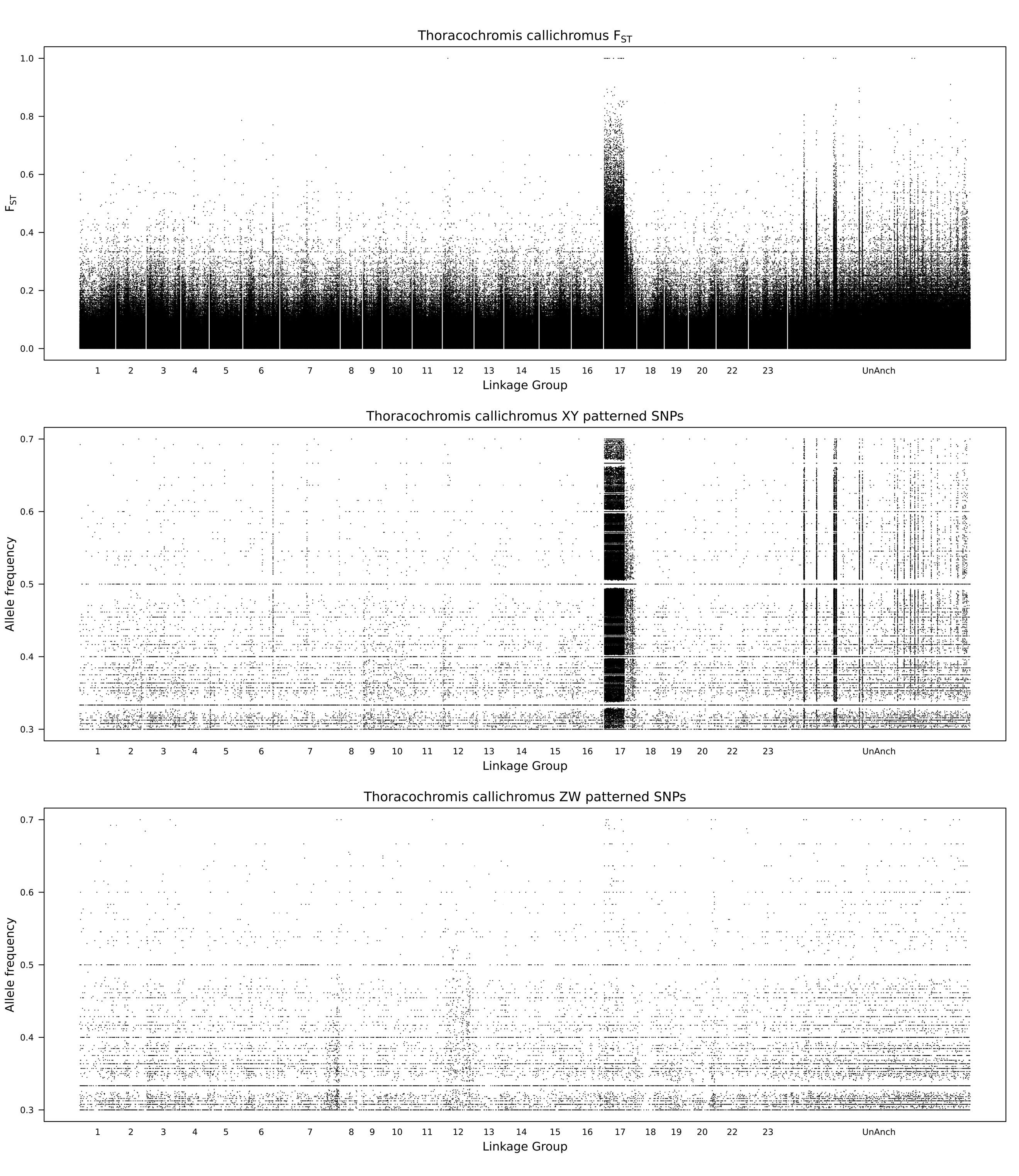
**

a

**
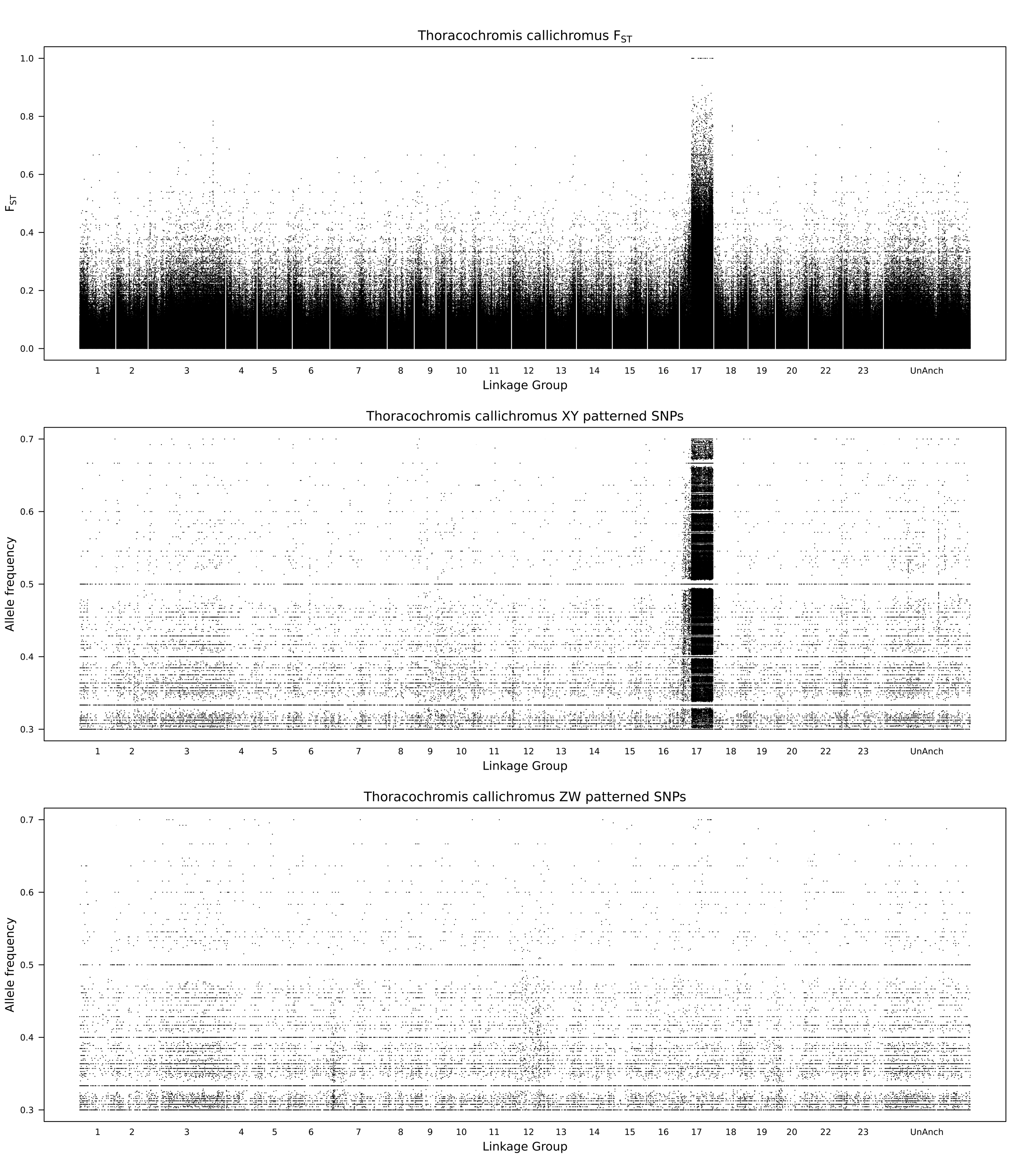
**

b

**
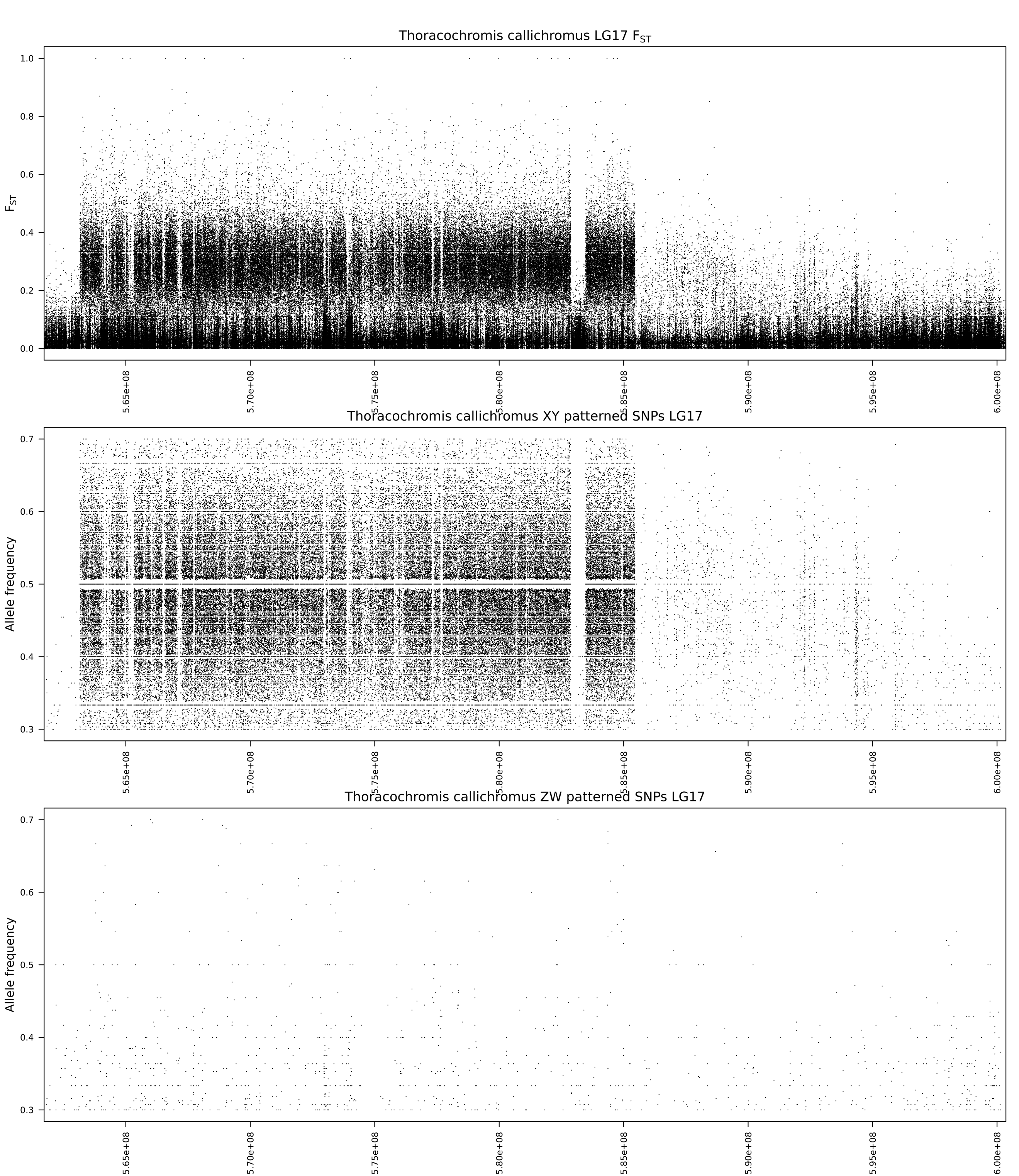
**

Position on chromosome (running genome size)

c

d

**Supplemental Figure 9.** *F*_ST_ and sex-patterned SNP plots for *Thoracochromis callichromus.* a) whole genome plot against *M. zebra* reference, b) whole genome plot against *O. niloticus* reference, c) single chromosome *F*_ST_ and sex-patterned SNP plots against *M. zebra* reference, d) single chromosome sex-patterned SNP density per 100kb window plots against *M. zebra* reference.


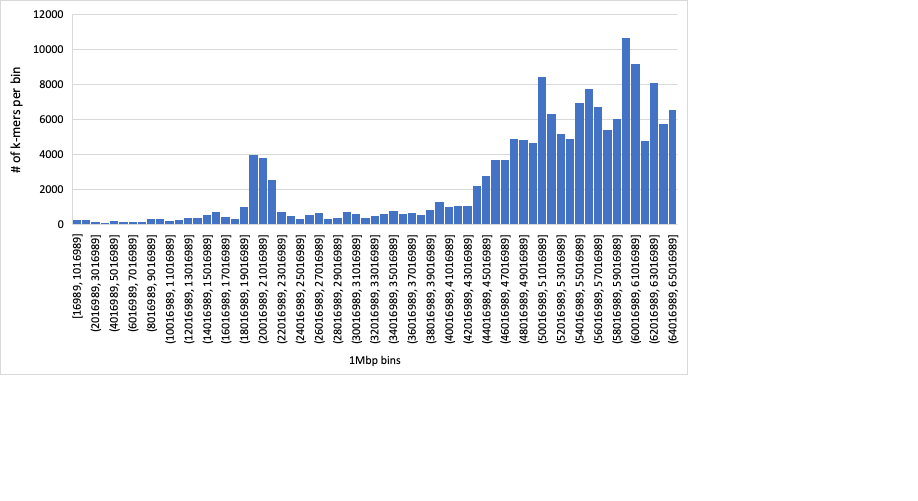


**Supplemental Figure 10**. Shared male-specific k-mers between *P. philander* Thamalakane River and *P. philander* Lake Chila plotted by number of k-mers per 1Mbp bin along the length of LG7.


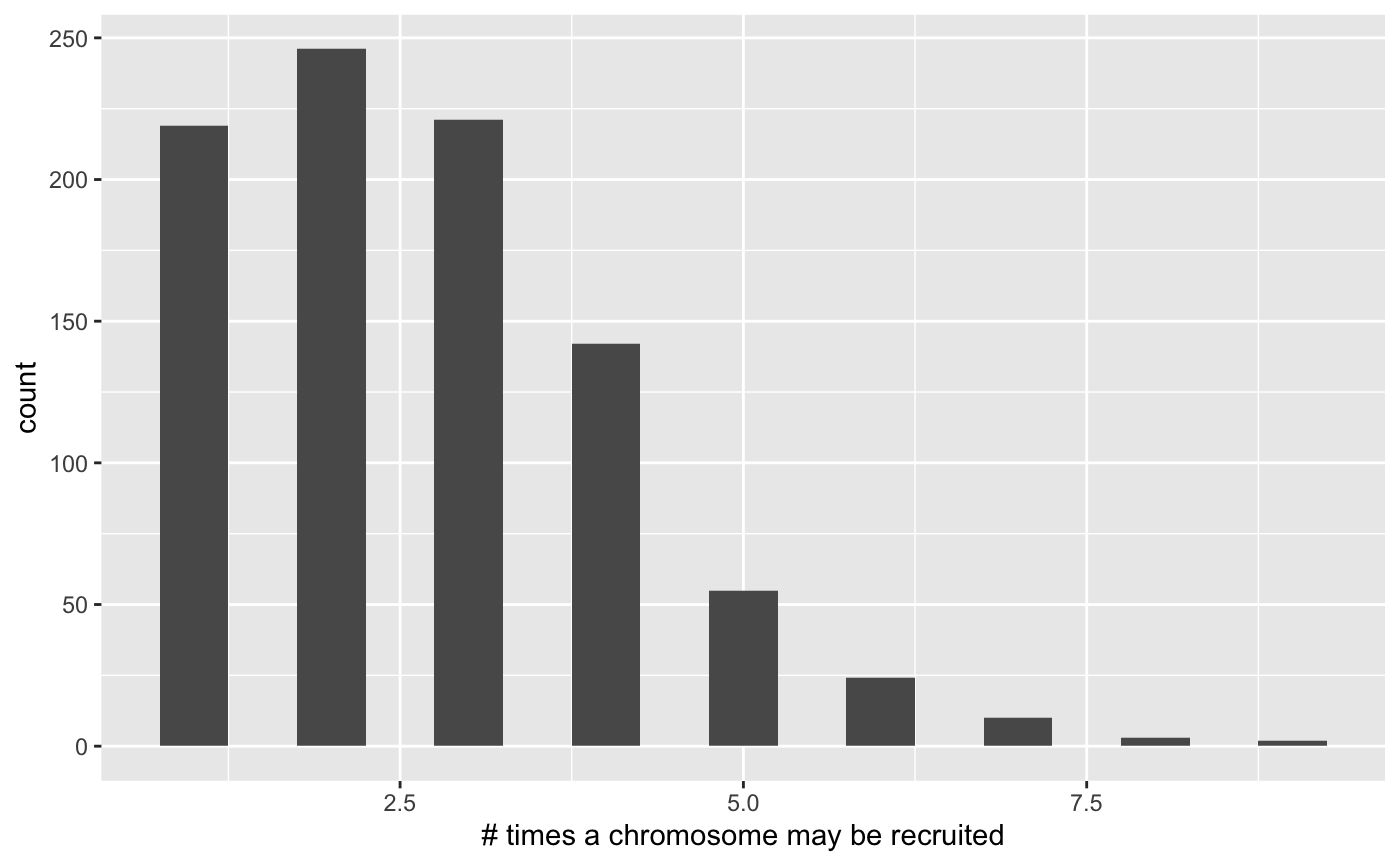


a

b


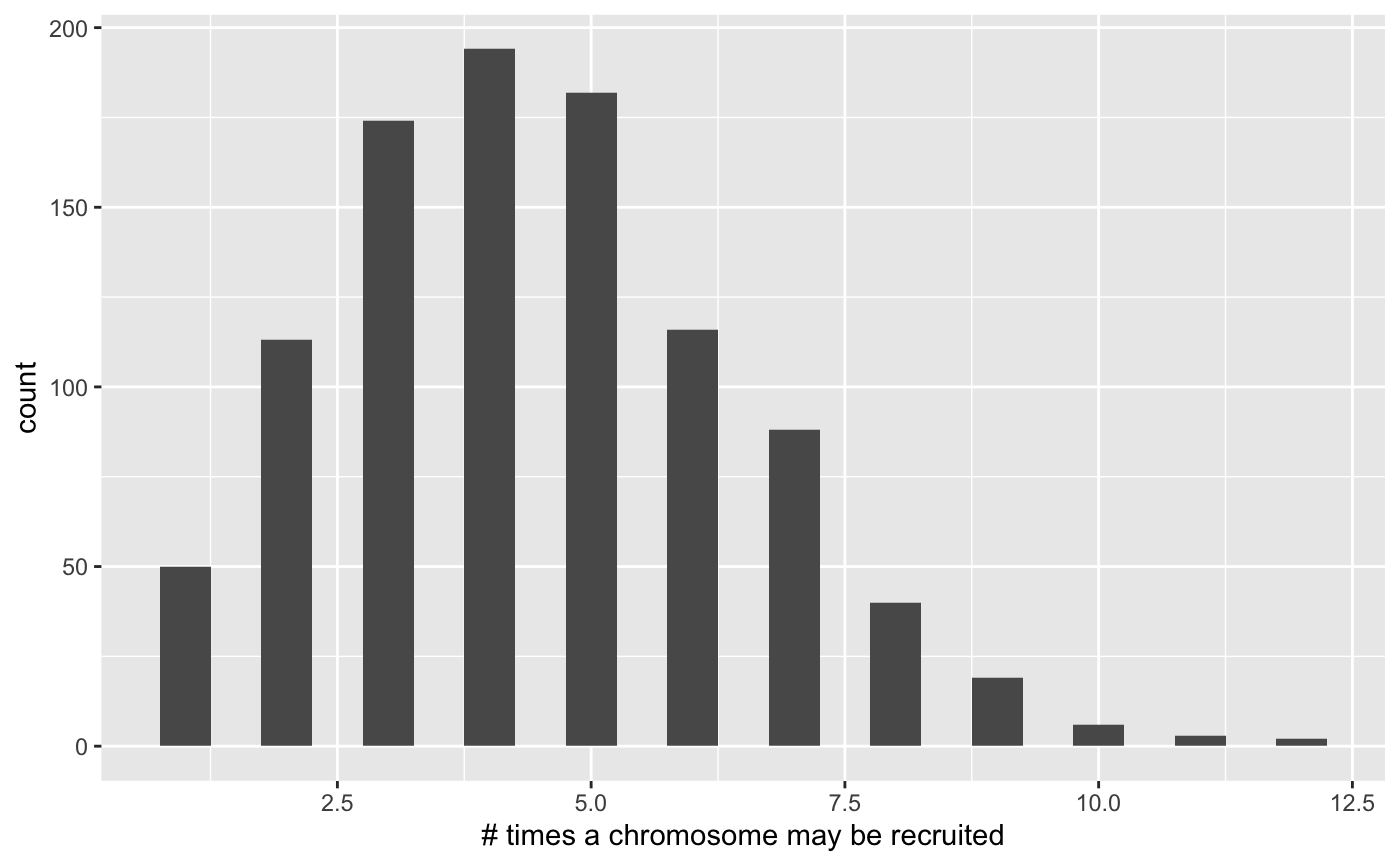


**Supplemental Figure 11**. Monte Carlo simulation of the expected distribution of 51 instances of novel sex chromosomes, accounting for the size differences among the 23 cichlid chromosomes. a) LG5 (7 instances observed) , p-val = 0.0043 b) LG7 (6 instances observed), p-val = 0.2444.
